# Brucellosis: epidemiology, pathogenesis, diagnosis and treatment–a comprehensive review

**DOI:** 10.1080/07853890.2023.2295398

**Published:** 2024-01-02

**Authors:** Kamal A. Qureshi, Adil Parvez, Nada A. Fahmy, Bassant H. Abdel Hady, Shweta Kumar, Anusmita Ganguly, Akhtar Atiya, Gamal O. Elhassan, Saeed O. Alfadly, Seppo Parkkila, Ashok Aspatwar

**Affiliations:** a Department of Pharmaceutics, Unaizah College of Pharmacy, Qassim University, Unaizah, Saudi Arabia; b Faculty of Medicine and Health Technology, Tampere University, Tampere, Finland; c Department of Biotechnology, School of Chemical and Life Sciences, Jamia Hamdard University, New Delhi, India; d Center for Microbiology and Phage Therapy, Biomedical Sciences, Zewail City of Science and Technology, Giza, Egypt; e Faculty of Applied Health Science, Galala University, Suez, Egypt; f Department of General Medicine, All India Institute of Medical Sciences, Bhopal, India; g Department of Biotechnology, Pondicherry University, Puducherry, India; h Department of Pharmacognosy, College of Pharmacy, King Khalid University (KKU), Abha, Saudi Arabia; i Department of Pharmacy Practice, Unaizah College of Pharmacy, Qassim University, Unaizah, Saudi Arabia; j Fimlab Ltd., Tampere University Hospital, Tampere, Finland

**Keywords:** Brucellosis, zoonotic disease, *Brucella*, livestock and wildlife, public health threats, antibiotic-resistant strains

## Abstract

**Background:** Brucellosis is a pervasive zoonotic disease caused by various *Brucella* species. It mainly affects livestock and wildlife and poses significant public health threats, especially in regions with suboptimal hygiene, food safety, and veterinary care standards. Human contractions occur by consuming contaminated animal products or interacting with infected animals. **Objective:** This study aims to provide an updated understanding of brucellosis, from its epidemiology and pathogenesis to diagnosis and treatment strategies. It emphasizes the importance of ongoing research, knowledge exchange, and interdisciplinary collaboration for effective disease control and prevention, highlighting its global health implications. **Methods:** Pathogenesis involves intricate interactions between bacteria and the host immune system, resulting in chronic infections characterized by diverse clinical manifestations. The diagnostic process is arduous owing to non-specific symptomatology and sampling challenges, necessitating a fusion of clinical and laboratory evaluations, including blood cultures, serological assays, and molecular methods. Management typically entails multiple antibiotics, although the rise in antibiotic-resistant *Brucella* strains poses a problem. Animal vaccination is a potential strategy to curb the spread of infection, particularly within livestock populations. **Results:** The study provides insights into the complex pathogenesis of brucellosis, the challenges in its diagnosis, and the management strategies involving antibiotic therapy and animal vaccination. It also highlights the emerging issue of antibiotic-resistant *Brucella* strains. **Conclusions:** In conclusion, brucellosis is a significant zoonotic disease with implications for public health. Efforts should be directed towards improved diagnostic methods, antibiotic stewardship to combat antibiotic resistance, and developing and implementing effective animal vaccination programs. Interdisciplinary collaboration and ongoing research are crucial for addressing the global health implications of brucellosis.

## 1. Introduction

Brucellosis is a zoonotic disease that primarily affects both livestock and wildlife. This infectious condition has considerable public health implications and causes significant economic challenges, especially in regions with inadequate food safety measures, hygiene standards and veterinary care. Brucellosis transmission to humans occurs through ingesting contaminated animal products or direct contact with infected animals. The disease exhibits endemicity in regions such as the Middle East, Mediterranean, Central and South America [1].

*Brucella* spp. exploit host immune defences to establish chronic infections, leading to a spectrum of clinical manifestations ranging from fever, fatigue and joint pain to more severe complications, such as endocarditis and neurological disorders [2]. Diagnosis of brucellosis necessitates the use of clinical evaluations and laboratory examinations, including blood cultures, serological testing and molecular approaches. Nevertheless, diagnostic challenges arise due to the non-specific nature of the symptoms and difficulties in obtaining suitable samples for testing [3].

Treatment strategies typically involve a combination of multiple antibiotics; however, the emergence of antibiotic-resistant *Brucella* strains presents significant challenges [4]. Vaccination of animals that are potential carriers of bacteria, especially within livestock populations, is promising for controlling the spread of brucellosis [5].

Despite concerted efforts to manage and mitigate the spread of brucellosis, it continues to pose a significant challenge to public health. This study provides a complete overview of brucellosis, specifically emphasising its epidemiology, pathophysiology, diagnosis and treatment methods. It also presents the latest research on *Brucella* species, their host range, and their transmission modes.

Finally, the review emphasises the need for sustained research and interdisciplinary collaboration among public health officials, healthcare providers and veterinary experts to develop effective strategies to control and prevent brucellosis [6]. This provides a comprehensive understanding of brucellosis and its impact on global health.

## 2. Epidemiology of brucellosis

### 2.1. Global prevalence

Brucellosis is a zoonotic infection caused by various *Brucella* species, affecting both humans and animals, including cattle, dogs, sheep and goats [7, 8]. Recent studies reveal a higher global incidence than previously estimated, with 1.6–2.1 million new human cases annually [9, 10]. Resource-limited regions, such as the Mediterranean, Middle East, Central Asia and certain parts of Africa, report elevated incidence rates [7]. Iran, Kyrgyzstan, Tajikistan, Kazakhstan, Azerbaijan, Turkmenistan, Armenia and Uzbekistan are among the countries with the highest reported incidences of brucellosis [11, 12].

### 2.2. Regional Epidemiology

#### 2.2.1. Regional epidemiology–California, United States of America

In Latin America, Mexico and Peru have reported many cases [13]. A study conducted by Fritz et al. on the epidemiology of brucellosis in California found that the disease is particularly prevalent among older Latino men, with a significant link to the consumption of unpasteurised Mexican-style soft cheese and *B. melitensis* was the most common species detected in cases. There were 492 cases reported in California from 1993 to 2017, underscoring the health risks of brucellosis. This study emphasises the importance of public health initiatives to inform the Latino community, especially the older population, about the risks associated with importing and consuming unpasteurised dairy products, particularly those from Mexico [14].

#### 2.2.2. Regional epidemiology–Europe

In the 28 EU countries, the annual incidence rate for 2017–2018 was 0.09 per 100,000 people [15]. The European Food Safety Authority (EFSA) noted a decline in brucellosis cases from 735 in 2008 to 352 in 2011, highlighting successful intervention measures [13].

In Europe, *Brucella canis* has emerged as a cause of canine brucellosis, indicating a zoonotic threat to public health. The lack of comprehensive surveillance and awareness of *B. canis* among veterinarians and dog owners complicates disease management. The current diagnostic tools for detecting *B. canis* infection are insufficient in sensitivity and specificity, underscoring the need for better diagnostic methods. The lack of universal reagents and standards for serological tests adds to the challenge of accurately diagnosing this infection. To address these issues, this study emphasises the importance of developing awareness materials, profession-specific guidance and enhanced diagnostic techniques to curb the spread of *B. canis* and increase awareness among the public and professionals [16].

#### 2.2.3. Regional epidemiology–Bosnia and Herzegovina

Between 2008 and 2018, 263 cases were studied in Bosnia and Herzegovina, decreasing from 102 in 2008 to 3 in 2018. The findings of this study regarding epidemiological characteristics align with the data from other global studies. Specifically, there was a notable male predominance; the most affected age group was between 25 and 49 years, and most patients either hailed from rural settings or had previous exposure to animals [13, 15, 17, 18].

#### 2.2.4. Regional epidemiology–Turkey

A study was conducted in Turkey to investigate the prevalence of brucellosis in children. The primary risk factor identified was occupational exposure, with 71.1% of the studied families engaging in animal breeding. Additionally, having a family member previously diagnosed with brucellosis accounted for 15.6% of the total risk. The study also emphasised that consuming raw milk and dairy products, such as cheese, is the primary transmission route in most instances. These findings are consistent with previous studies conducted in other regions of Turkey [19].

#### 2.2.5. Regional epidemiology–Iran

Brucellosis is present in most parts of Iran, with 80,000 cases reported annually since 1989. It has been reported in Iran that healthcare workers are accidentally exposed to *Brucella* strains during routine animal vaccination programs [20]. Brucellosis incidence in Iran varies by region and has decreased in recent years. Males aged 25–29 years are more commonly affected by the disease, with western provinces reporting higher prevalence. The seasonality of brucellosis cases is notable, with spring months seeing increased diagnoses. Occupational risks for healthcare workers, including accidental exposure during animal vaccination programs, highlight the need for targeted prevention strategies. *Brucella melitensis* biovar 1 remains the dominant causative agent in Iran, and risk factors include the consumption of unpasteurised dairy products and living in rural areas. Efforts to control and manage brucellosis in Iran require a multifaceted approach that addresses regional variations and occupational exposures [21–24].

#### 2.2.6. Regional epidemiology–Jordan

In Jordan, brucellosis is prevalent among young adults in rural areas and those working in livestock-related occupations, particularly during the spring and summer. Al-Amr et al. revealed variations in seropositivity rates, with occupational exposure being a significant risk factor. This research also highlights the substantial health burden of brucellosis in Jordan, exceeding that in North America and Western Europe, with 31.1% of febrile illnesses in Jordan attributed to the disease. These findings are crucial in informing and enhancing disease control and prevention strategies, offering valuable insights into the epidemiology of brucellosis in Jordan and contributing to reducing its impact [25].

#### 2.2.7. Regional epidemiology–India

A study by Holt et al. [26] revealed that brucellosis, a zoonotic disease caused by *Brucella* species, is endemic in rural areas of India, with a seroprevalence of 15.1% (95% CI: 15.9–19.8%). This finding emphasises the disease’s prevalence in regions where agriculture and livestock farming are common, facilitating disease transmission due to close human–animal interaction. Seroprevalence, denoting the presence of *Brucella* antibodies in individuals’ blood, highlights substantial exposure within the rural population. Moreover, the study’s 95% confidence interval underscores the statistical reliability of this seroprevalence estimate. In conclusion, Holt et al.’s research underscores brucellosis as a significant health concern in rural India, necessitating effective control measures and increased community awareness to address this zoonotic disease’s impact [26].

#### 2.2.8. Regional epidemiology–Punjab, Pakistan

In Punjab, Pakistan, a study by Nawaz et al. on the epidemiology of brucellosis revealed a seroprevalence of 13.13%, with higher rates in males aged 25–40. Risk factors were lack of education, involvement in farming, keeping animals at home, animal slaughter and consumption of raw milk. This study further emphasises the necessity of raising awareness regarding disease transmission and risk factors among individuals with direct animal exposure, particularly livestock farmers. Furthermore, it underscores the importance of avoiding unpasteurised dairy products to mitigate the spread of this often underestimated zoonotic disease, which has a high regional morbidity rate [27].

#### 2.2.9. Regional epidemiology–China

A study in China examined the epidemiological features, morbidity and endemic nature of human brucellosis and observed a notable increase in the population [28]. Four-year study revealed divergent trends in the incidence of brucellosis across China, with a nationwide average annual incidence of 3.0 per 100,000 people. While the rate substantially decreased in Xinjiang, it more than doubled in Inner Mongolia, contributing to the higher incidence rate in Northern China. Notably, males aged 45–64 in this region are more than twice as likely to be affected by their female counterparts [29].

#### 2.2.10. Regional epidemiology–Mongolia

A study found a declining incidence but increasing seroprevalence of human brucellosis in Inner Mongolia from 2012 to 2016, with genetic data pointing to both sporadic cases and cross-infections potentially exacerbated by long-term livestock trade [30]. Notably, Mongolia ranks second globally in incidence, whereas Syria has the highest annual prevalence.

#### 2.2.11. Regional epidemiology–sub-Saharan Africa

Brucellosis is endemic to many regions of the world, including sub-Saharan Africa. According to the literature published between 2010 and 2019, the prevalence of brucellosis in livestock ranged from 0.2% to 43.8% in cattle, 0.0% to 20.0% in goats, and 0.0% to 13.8% in sheep. In humans, the prevalence of brucellosis in the sub-Saharan African region ranges from 0% to 55.8%, highlighting the significant presence of brucellosis infection in this area [31].

## 3. Transmission of brucellosis

The prevention and control of brucellosis is of paramount significance, and a thorough understanding of its mode of transmission is indispensable in achieving this objective. Brucellosis can be transmitted to humans through several paths.

### 3.1. Direct contact with infected animals

Brucellosis is primarily transmitted through direct contact with infected animals or their bodily fluids, including vaginal discharges, aborted materials and semen. Those who work closely with livestock, such as farmers, veterinarians and livestock handlers, are at a heightened risk of contracting the disease due to their frequent interactions with animals [9, 10, 32].

### 3.2. Consumption of contaminated products

Brucellosis can also be transmitted through the consumption of raw or unpasteurised dairy products from infected animals, including milk and cheese. The consumption of these contaminated food products can result in human infection, emphasising the importance of food safety practices to prevent the spread of the disease [7].

### 3.3. Inhalation of airborne agents

In certain occupational settings, such as slaughterhouses and meat processing facilities, the airborne transmission of *Brucella* bacteria can become a concern. It is possible for workers in these environments to inhale airborne agents, which may result in infection. This highlights the necessity of implementing effective workplace safety measures and utilising appropriate protective equipment [9, 10].

### 3.4. Occupational hazard

Human brucellosis poses a significant risk factor for occupational exposure, particularly for individuals in professions such as butchers, laboratory workers and hunters, who have direct contact with infected animals or their products. To mitigate this risk, it is essential to implement occupational health precautions [7].

Laboratory-acquired human brucellosis infections are not uncommon [13]. For example, 12 out of 48 healthcare workers tested positive for *Brucella* spp. in a hospital facility in Ankara, resulting in an infection risk of 8% per employee per year [19]. While person-to-person transmission is rare, it is crucial to recognise other potential sources of brucellosis transmission. These include blood transfusions and bone marrow transplants, underscoring the importance of antibody detection methods, especially in endemic areas [12, 13, 20].

Additionally, brucellosis can be transmitted through inhalation of aerosols, contact with contaminated skin, and colonisation of the udder by using contaminated milking equipment [12, 13, 20]. It is worth noting that brucellosis is considered a potential type B bioweapon [33]. Furthermore, the unhygienic processing of milk, milk products and meat has contributed to the spread of human brucellosis, highlighting its zoonotic nature [13, 33]. In conclusion, occupational exposure is a significant concern in brucellosis transmission. Professionals in specific fields need to be vigilant and take appropriate precautions to reduce the risk of infection, and public health measures should address these potential transmission sources.

### 3.5. Indirect transmission

Indirect transmission can occur through contact with contaminated materials or environments. People encountering surfaces or objects contaminated with *Brucella* can become infected. Proper sanitation and hygiene practices are essential in reducing the risk of indirect transmission [9, 10].

### 3.6. Intrauterine transmission

Brucellosis, although rarely transmitted, can be passed from an infected mother to her unborn child during pregnancy, underscoring the significance of prenatal care and monitoring for pregnant individuals with brucellosis [9, 10, 32]. Therefore, a thorough understanding of the various modes of transmission is crucial to prevent its spread. Implementing effective preventive measures, such as vaccinating livestock and promoting public health education, is imperative in controlling this zoonotic disease.

## 4. Trends and seasonal variations

The seasonal trends showed the highest prevalence of brucellosis from March to June. Spring showed a marked distribution of brucellosis in areas where the disease is endemic [15]. A study by Delam et al. conducted from 2015 to 2020 utilising the Cochran–Armitage test, revealed that the average annual incidence of the disease was 8.94 per 100,000 individuals. The study also found a significant decrease in the incidence rates, dropping from 26.83 per 100,000 people in 2015 to 1.83 per 100,000 people in 2020. The Cochran–Armitage test confirmed that this reduction in incidence was statistically significant [34].

## 5. Species and biovars

The *Brucella* genus comprises six species, each categorised by its principal host: *B. melitensis* (sheep and goats), *B. abortus* (cattle), *B. suis* (pigs), *B. ovis* (sheep), *B. canis* (dogs) and *B. neotomae* (wood desert rats). *B. melitensis* is the most virulent bacterium in humans [10, 35]. *B. abortus* is found worldwide in cattle-raising regions, except Japan, Canada and some European countries [12]. In recent years, several new species have been successfully isolated, including *B. inopinata* (from humans), *B. pinnipedialis*, *B. ceti* (from aquatic animals) and *B. microti* (from the common vole), expanding the count to 10 species [36].

Studies have shown that each *Brucella* species comprises multiple biovars. *B. abortus* consists of eight different biovars (1–7 and 9), *B. melitensis* has three biovars (1–3) and *B. suis* has five biovars (1–5). Other *Brucella* species have not yet been differentiated into biovars [37, 38]. In a study by Liu et al., 107 human isolates of *Brucella*, identified explicitly as the *B. melitensis* species and predominantly as biovar 3, were subjected to an MLVA-16 assay to explore their genetic diversity. This assay classified these isolates into 75 unique MLVA-16 genotypes. Intriguingly, 54 of these genotypes represent unique, epidemiologically unrelated and sporadic cases of brucellosis. In contrast, the remaining 21 shared genotypes among two to four strains indicated cross-infections and multiple outbreak events. Moreover, substantial genotype overlap was observed with strains from Kazakhstan, Mongolia and Turkey, which are the key members of the Grassland Silk Road. The extensive trade of small ruminants, mainly sheep, in these countries may have contributed to the regional spread of *Brucella* spp. [30].

## 6. Diagnostic challenges and public health response

Brucellosis poses numerous diagnostic challenges that significantly hinder public health initiatives. These difficulties are closely related to the extent of contact individuals have with infected animals or their products [9, 10]. Here are some crucial aspects to take into account:

- *Misdiagnosis and underdiagnosis*: One of the main obstacles in diagnosing brucellosis is the risk of misdiagnosis or underdiagnosis. The symptoms of the disease, such as fever, fatigue and joint pain, are non-specific and can be similar to those of various other illnesses. As a result, healthcare providers may either overlook the diagnosis or confuse it with other conditions, leading to delayed appropriate treatment.
- *Resource-poor settings*: Diagnostic inaccuracies, particularly misdiagnosis and underdiagnosis, are notably prevalent in regions with limited access to advanced diagnostic equipment and healthcare infrastructure. Consequently, the disease burden in such resource-poor settings may be considerably underestimated, resulting in inadequate public health responses.
- *Overestimated case numbers*: The paradoxical nature of diagnosing brucellosis is such that the difficulty in doing so can lead to an overestimation of case numbers in regions where healthcare systems rely on less specific diagnostic methods. This overestimation can in turn foster heightened risk perceptions and unwarranted panic among the general population.

In response to these challenges, several crucial measures need to be implemented:

- *Improved diagnostic methods*: The development of reliable diagnostic tests for brucellosis is imperative, as current methods are often inaccurate and slow. Research and development initiatives should focus on creating tests that can accurately differentiate brucellosis from other febrile illnesses and are suitable for use in resource-limited settings. These tests should also be easily accessible to improve early diagnosis and treatment of the disease.
- *Enhanced surveillance*: The implementation of comprehensive surveillance systems is crucial for the purpose of closely monitoring the occurrence and geographic proliferation of brucellosis. This entails identifying outbreaks at an early stage and gaining insight into the disease’s epidemiological patterns within particular demographics.
- *Increased awareness*: Brucellosis, a zoonotic disease with significant public health implications, requires increased awareness and education among health professionals, particularly those working in endemic regions. Enhanced understanding of the clinical manifestations and diagnostic challenges associated with the disease is crucial for accurate and timely diagnoses, which is essential for effective public health responses. Improved diagnostics, strengthened surveillance and increased awareness among healthcare providers are vital components in mitigating the impact of brucellosis, especially in resource-poor settings where the burden is often high. These efforts are essential for controlling the spread of the disease and minimising its impact on affected populations.

## 7. Virulence factors and pathogenesis

Regarding virulence factors, *Brucella* lacks classical factors such as exotoxins, cytolysins and exoenzymes. Its pathogenesis is attributed to unique factors, such as lipopolysaccharide (LPS), type IV secretion system (T4SS) and the BvrR/BvrS system. These factors facilitate interactions with host cells, the formation of *Brucella*-containing vacuoles (BCVs), and interactions with the endoplasmic reticulum (ER) during bacterial multiplication [39–41].

The pathogenesis of brucellosis is intricate and involves bacterial invasion of host cells, immune evasion and chronic infections. *Brucella* uniquely penetrates and persists within host cells, such as macrophages, and uses strategies to bypass host immune defences, leading to prolonged infection. Symptoms in humans include fatigue, fever, generalised discomfort, and more severe manifestations, such as arthritis, osteomyelitis, endocarditis and meningoencephalitis [39–41]. *Brucella* is an adept intracellular pathogen that can survive and replicate within the host cells, evading the immune system. They inhibit phagocytosis, reduce bactericidal activity, diminish endotoxic reactions and impede antigen presentation [39–41].

The pathogenesis of *Brucella* extends to its survival and multiplication within phagocytic and non-phagocytic cells, its ability to manipulate host cell processes, disrupt phagocyte function, inhibit phagocytosis and prevent host cell apoptosis. It modulates the host immune response by targeting signalling pathways involved in innate immunity, such as the degradation of the TLR signalling adapter MAL [39–41]. *Brucella*’s resilience in various environments, including water, soil, dairy products and meat, further contributes to its pathogenesis and transmission [39–41].

## 8. Clinical manifestations

The clinical manifestations of brucellosis vary significantly, making the diagnosis challenging. Symptoms range from flu-like illnesses to more severe complications involving multiple organs. The non-specific nature of symptoms and the difficulty in obtaining samples for laboratory testing contribute to challenges in diagnosing the disease. Haemorrhagic anaemia is an important clinical manifestation of brucellosis in children. *Brucella* infections can lead to microangiopathic haemolytic anaemia and severe thrombocytopenia in children. The ability of *Brucella* to change from a non-haemolytic to a haemolytic phenotype may influence its pathogenicity and contribute to the correlation between acute brucellosis and haemolytic anaemia in humans. The expression of haemolysin genes in *Brucella* may have accumulated mutations during growth, resulting in the repair of the default genes and the ability to express haemolysin, which can affect pathogenicity. However, a conclusive explanation for the development of haemolytic anaemia during *Brucella* infection is still missing. The presence of haemolysin genes and haemolytic anaemia in humans has been reported [42].

### 8.1. Symptoms and complications associated with brucellosis

Brucellosis in humans is a multifaceted disease that affects various organs with a diverse range of symptoms ranging from mild to severe. This complexity often leads to misdiagnosis. If untreated, it may transition to the chronic phase, increasing the risk of disability [43]. The incubation period ranged from five days to 6 months [44]. Upon ingestion, *Brucella* bacteria are carried by macrophages to lymphoid tissues, spread through the lymphatic system, and can potentially multiply in multiple organs, causing localised and systemic infections [45].

The symptoms can persist for months or years in chronic cases [45], making a comprehensive medical and dietary history essential for accurate diagnosis, especially in non-endemic regions where infection can occur from consuming imported contaminated food [46]. Brucellosis presents symptoms such as headaches, recurring fever, migratory joint pain, muscle pain, weakness, loss of appetite, fatigue, general discomfort, sweating, vomiting, diarrhoea, abdominal pain and even miscarriage [41]. Complications such as sacroiliitis, osteomyelitis, spondylodiscitis, septic arthritis and epidural abscesses may arise [47]. Rarely, brucellosis is linked to conditions such as hepatic abscesses, granulomas, spontaneous bacterial peritonitis, ventriculoperitoneal shunt infection and immune thrombocytopenic purpura [48–52]. Respiratory symptoms and neurological manifestations, including Guillain-Barre syndrome, can also occur [53, 54]. Death is rare, with cases reporting a unique unpleasant odour in patients’ sweat [55, 56].

Physical examination findings may appear normal, but signs such as lymphadenopathy, splenomegaly and hepatomegaly may be present [45]. Epididymoorchitis and endocarditis are uncommon complications, with the latter being the primary cause of death related to brucellosis [57, 58]. Ocular and skin manifestations such as uveitis, keratoconjunctivitis, iridocyclitis, optic neuritis, cataracts, maculopapular eruptions, erythema nodosum, abscesses and panniculitis have also been reported [59–62].

The CDC and the WHO do not precisely define chronic brucellosis. Generally, symptoms persist for over a year after the initial diagnosis [46]. Patients with chronic brucellosis exhibit either a focal complication with objective evidence of infection or persistent symptoms without objective signs of infection, manifesting as general malaise and psychiatric complaints, such as depression and anxiety [4, 63].

### 8.2. Relapse after treatment

The incidence of relapse after treatment ranges from 5% to 15%. Typically, relapses are observed within six months after treatment completion, although they can occur up to 12 months later [64]. The multivariate model identified several factors as independent predictors of relapse. These factors include a temperature of 38.3 °C or higher, experiencing symptoms for less than 10 days before starting treatment, and testing positive for *Brucella* in blood cultures [65]. Distinguishing between relapse and reinfection can pose challenges, particularly in regions where individuals are continuously exposed to infectious agents [64]. Relapses can be attributed to various causes, such as an insufficient antibiotic treatment plan, a shorter duration of antibiotic therapy than required, nonadherence to the prescribed regimen, or localised areas of infection. Relapses rarely occur because of antibiotic resistance [66].

### 8.3. Laboratory diagnosis and its challenges

Humans can act as sporadic hosts for *Brucella* infection, primarily due to a virulent strain, which is *B. melitensis*, *B. abortus*, *B. suis* and *B. canis.* Identifying human cases of brucellosis relies heavily on microbiological analysis because of the variability and lack of specificity of the symptoms associated with the disease [67]. Diagnostic tools for brucellosis include *blood culture*, *serological assays* and *molecular methods*. Each method has advantages and limitations, such as sensitivity, specificity and time required for accurate results. The choice of diagnostic method depends on clinical presentation and available resources. There are three primary techniques used for microbiological identification of human brucellosis: blood cultures, serology assays and molecular assays. Here, we summarise recent advancements in evaluating diagnostic techniques, their clinical value, and their respective advantages and disadvantages [68, 69].

#### 8.3.1. Blood cultures

Peripheral blood cultures are crucial for confirming human brucellosis, especially during bacteraemia. However, their sensitivities were inconsistent, ranging from 10% to 90%. Patients with early-stage brucellosis often have low-level persistent bacteraemia detectable in multiple blood samples. However, as the infection advances, the bacterial concentration in the blood drops, creating an unpredictable bacteraemia pattern and increasing bacterial isolation from blood specimens [70].

*Brucella* may intermittently re-enter the bloodstream, increasing the chances of clinical recurrence and spreading to other areas. Despite its generally low virulence in humans, *Brucella* spp. can be recovered from mildly symptomatic or even afebrile patients. This underscores the necessity of obtaining blood cultures from suspected brucellosis cases, even without fever [69].

Several factors affect the effectiveness of blood culture techniques for *Brucella* detection, including its long generation time and reduced CO_2_ emissions. For enhanced recovery, it is suggested to incubate the inoculated medium for four weeks and perform blind subcultures of seemingly negative blood culture media. Despite its effectiveness, this method is costly, labour-intensive and significantly delays the diagnosis.

Shorter incubation periods (3–7 days) have been explored, showing varying successes in detecting circulating *Brucella*. However, the risk of prematurely discarding vials containing viable *Brucella* cannot be ignored, highlighting the importance of carefully assessing the sensitivity and detection time of blood culture systems [71]. Various blood culture techniques have been employed.

- *Manual monophasic approach.* Patient blood samples were incubated in culture vials at 35 °C and checked for bacterial and fungal growth. However, *Brucella*’s slow growth often went undetected, leading to premature vial disposal within 5–7 days. To improve *Brucella* detection, vials were kept longer, and blind subcultures were conducted when brucellosis was suspected.
- *Manual biphasic approach*
  - *Castañeda flask:* Ruiz-Castañeda proposed a biphasic flask to avoid repeated blind subcultures, save time and labour, and minimise the risk of laboratory-acquired brucellosis. Despite its non-specificity to *Brucella*, it remains popular in regions with limited resources owing to its affordability and practicality [72]. Proper identification is essential for confirming the presence of *Brucella* spp.
  - *TUMS medium:* A variant in the Castañeda flask medium, Tehran University of Medical Sciences (TUMS) medium, expedites *Brucella* identification. It uses a solid urea agar substrate to note the colour change of the pH indicator, indicating *Brucella*-positive urease activity.
  - *Hémoline medium:* A study on Hémoline, a commercial biphasic blood culture medium, showed a five-day detection period for *Brucella*. However, in 23.5% of the cases, detection was delayed beyond seven days.
- *Lysis-based blood cultures.* In this method, white blood cells are lysed before seeding onto a solid medium. This is crucial, as *Brucella* does not circulate freely in the blood but is engulfed by specific white blood cells. This engulfment often leads to bacterial death, lowering culture sensitivity and prolonging the detection time. Lysing white blood cells releases live *Brucella*, which is then transferred to solid media, enhancing the likelihood and speed of detecting viable *Brucella* organisms in culture.
- *Blood clot medium*. The procedure involves collecting a blood sample, allowing coagulation, and separating the serum by centrifugation for serodiagnosis. The clot was shaken vigorously to break it apart, and the material was spread on solid agar media for cultivation. Despite its rational basis, this approach has limited published data and has shown inconsistent results, necessitating further research and evaluation to optimise this method for brucellosis detection [73].
- *Automated blood culture method*. Recent advancements in CO_2_ monitoring and liquid culture media have notably enhanced the diagnosis of *Brucella* bacteraemia. These modern bacteriological techniques boost the sensitivity of *Brucella* cultures, decrease the detection time, and make the diagnostic procedure more efficient. Automated systems employing these techniques can process numerous blood culture bottles, reduce media contamination, and ensure safe bacterial management.

Despite the conventional practices of extended incubation and intermittent subculturing, modern blood culture techniques promise increased sensitivity and faster detection. If used early in the infection, these automated techniques can identify acute brucellar infections within a standard one-week incubation period, negating the need for sub-culturing presumed negative vials. However, some patients may require prolonged incubation and terminal subcultures, especially in long-duration or focused infection cases.

Modern technologies provide quicker results than traditional culture methods, especially for slow-growing organisms, such as *Mycobacterium tuberculosis*.

- *Radiometric detection (BACTEC 460TB system):* This method uses a radiometric approach to detect CO_2_ produced by the metabolism of the bacterium. The BACTEC 460TB system is widely used for rapidly detecting mycobacteria but has been largely replaced by non-radiometric systems because of concerns regarding radioactive waste disposal.
- *Fluorescence and colorimetric methods (e.g. BACTEC MGIT 960 system):* Instead of radiometric detection, these systems rely on fluorescent or colorimetric indicators that change in response to O_2_ consumption or CO_2_ production during bacterial growth.
- *BacT/alert system:* This continuous monitoring system detects CO_2_ production using colorimetric sensors.
- *Bactec 9000:* Similar to the BACTEC 460TB system, this system uses fluorescence instead of radiometry.
- *Infrared technology:* Some newer systems use infrared technology to detect CO_2_ production to indicate bacterial growth.

All these technologies can reduce the time required for detection compared to traditional culture methods. The choice of method depends on laboratory requirements, available resources and specific applications. Comparative studies suggest that the automated Bactec system is more effective for recovering *Brucella* in blood cultures, outperforming the isolator microbial tube in terms of overall susceptibility and detection time. Nonetheless, further research is essential to comprehensively assess the performance of other available blood culture systems [74]. In conclusion, the appropriate diagnosis of brucellosis depends on various patient- and method-related factors, necessitating a careful approach to blood culture techniques for optimal *Brucella* detection and identification. These factors are summarised in Table 1.

**Table 1.** Factors affecting brucellosis detection in blood culture.

Characteristics	Related factors
Microbiological	*Brucella* spp. involved.
Patient	Age of the patient, systemic (involving multiple organs) or focal (localised to specific organs), differentiating between a first-time infection and a relapse of a previous infection, prior or ongoing antibiotic treatment.
Culture technique and specimen	Volume and number of specimens, rate of detection, susceptibility of culture, period of incubation, and periodicity of blind subcultures (subculturing of negative blood cultures at regular intervals can help identify slow-growing or low-level bacterial growth).

##### 8.3.1.1. Advantages

Patients with acute brucellosis can detect the causative organism within the standard seven days incubation period, eliminating the need for subculture. In many laboratories, automated blood culture systems (BacT/Alert, BACTEC 9000 series, Vital, and ESP) for brucellosis have assumed the position of traditional blood culture systems. These methods appear to reduce the time (∼3 days) required to identify these organisms in blood and other bodily fluids. Nucleic acid amplification assays, hybridisation tests and matrix-assisted laser desorption ionisation-time of flight mass spectrometry (MALDI-TOF) have advanced the identification and classification of *Brucella* species. These methods provide a rapid, accurate and safe means of identifying and classifying recovered *Brucella* isolates. Blood cultures are more helpful than serology during disease recurrence because the latter is already positive at relapse [75].

##### 8.3.1.2. Disadvantages

The sensitivity of blood cultures differs according to the laboratory protocol used to obtain the cultures and how actively they are collected. Fifteen to seventy percent of cases showed a positive culture rate. MALDI-TOF mass spectrometry technology offers several advantages, including relatively low cost per detected bacterium. However, it is worth noting that this technology is still expensive, which limits its widespread availability, particularly in countries where brucellosis is prevalent [75].

#### 8.3.2. Serological assays

In contrast to molecular and culture-based detection methods, serological diagnostics for brucellosis do not directly identify the presence of living bacteria or their DNA sequences in bodily fluids or tissues. Instead, the serological method relies on an indirect approach of examining the patient’s immune response for the presence of antibodies that indicate prior exposure or contact with the *Brucella* pathogen. It measures the presence of specific antibodies such as IgM and IgG in patients’ serums. Detection of these antibodies provides evidence of previous or ongoing brucellosis infection. The clinical application of the available serodiagnostic tests for brucellosis in humans is outlined in Table 2.

**Table 2.** Serodiagnostic assays for the diagnosis of human brucellosis [73, 69, 71, 76, 77].

Diagnostic assay	Principle	Recommended use	Merits	Demerits
Buffered Acidified Plate Antigen Test (BAPAT)	Detection of anti-*Brucella* antibodies in sheep serum samples	Screening test for brucellosis diagnosis in sheep herds	High sensitivity as a screening test for brucellosis	Lower specificity compared to other tests due to non-specific agglutinins
Rose Bengal Plate Test (RBPT)	Plate agglutination test detects agglutinating and non-agglutinating antibodies	Screening test for brucellosis diagnosis in sheep herds	Quick, cheap and simple Higher specificity compared to BAPAT due to pH inhibition of non-specific agglutinins	Lower sensitivity compared to BAPAT
Milk Ring Test (MRT)	Detection of anti-*Brucella* antibodies in sheep milk samples.	Screening test for brucellosis diagnosis in sheep herds.	Easy application and high-sensitivity	Less sensitive in detecting antibodies in milk with low concentrations or fat clustering factors
Standard Tube Agglutination Test (STAT)	Total antibodies against S-lipopolysaccharide (S-LPS) on bacterial surfaces detected	Safe, popular, especially in acute cases.	Practical, efficient and cost-effective	They have limitations in detecting *B. canis* infection, leading to false negatives due to blocking of antibodies, cross-reactivity and non-specific agglutination.
2-Mercaptoethanol test	Chemical inactivation of the IgM pentamer’s agglutinating properties by 2-mercaptoethanol	Monitoring the effectiveness of antimicrobic medications in patients who have already received a diagnosis; early identification of treatment failure	Confirmatory test–elimination of IgM confounder	Toxicity of 2-mercaptoethanol
*Brucella* Coombs Gel Test	Expansion of STAT	Sensitive to chronic infections and relapses.	Quick and straightforward (2 h)	Labour-intensive and time-consuming
Complement Fixation Test (CFT)	IgG1 isotype antibody detection by complement fixation	Useful for the serological diagnosis of zoonosis in obliteration operations	Sensitive	Due to its technical difficulty and issues with its standardisation, it is not frequently employed in human infection
Immunocapture agglutination test (*Brucella* Capt test; Vircell, Granada, Spain)	Detection of IgG, IgM and non-agglutinating antibodies to the three smooth O-polysaccharide-containing *Brucella* species in a single step	Confirmatory test, patient monitoring after treatment	Performance is equivalent to that of the Coombs test; however, it is quicker and simpler to complete	Individuals can vary greatly, and individuals who have relapsed may have a one-dilution decrease in titer.
Enzymatic linked immuno sorbent assay (ELISA)	The standard method of sensitising plates is with cytosolic protein antigens	Test of preference for complex, focused, and chronic patients. Neurobrucellosis and *B. canis* infection diagnosis	Compassionate, specific, rapid (4–6 h), simple. Detects total and individual specific Igs (IgG, IgM and IgA) when other tests are negative	Expensive and requires trained personnel
Immunofluorescence assay (IFA)	Antigens prepared from whole-cell preparations		Quick, accuracy equivalent to ELISA	Expensive equipment and manpower
Time-resolved fluorescent resonance energy transfer (TR-FRET) assay	Based on the transfer of energy between fluorophores-labelled antigens and antibodies (a donor and an acceptor). An anti-*Brucella* monoclonal antibody is labelled with a donor fluorophore and *Brucella* S-LPS	Simple to perform, robust and has excellent serodetection ability	Does not require washing and only a single 30-minute incubation time followed by fluorescence readout. They provide comparable performance to other diagnostic methods.	
Fluorescent polarisation immunoassay (FPA)	A fluorescent dye (labelled to an antigen or antibody) can be excited by polarised light, and difference in the rotational velocities is measured	Diagnosis of zoonosis in the dairy sector	Widely used in animals. Rarely used in human infections. Fast and straightforward method and equipment.	
Quantum dot (QD) immunochromatographic test system	Handheld QD immunochromatographic strip equipment	Point-of-care test and preliminary screening.	Rapid and simple. Specific, sensitive, reliable	

##### 8.3.2.1. Advantages

Despite these limitations, serological tests are crucial for diagnosing human brucellosis, particularly in endemic countries. Their cost-effectiveness and simplicity make them preferable to culture-based or nucleic acid amplification methods, particularly in resource-limited settings. In high-prevalence areas, serological testing is an accessible and practical diagnostic option, contributing to the timely diagnosis and management of brucellosis cases. The widespread availability and feasibility of serological tests bolster screening and surveillance efforts to ensure early detection and appropriate intervention [69, 71, 73, 76].

##### 8.3.2.2. Disadvantages

Various factors, such as early testing, blocking antibodies or the ‘prozone’ phenomenon, can lead to non-detection of brucellosis. Additives such as EDTA, 2-mercaptoethanol or anti-human globulin may overcome these issues, but serum agglutination tests remain unsuitable for follow-up due to sustained high titers [69, 71, 73, 76].

Analysing serological tests for brucellosis is challenging due to patient history variations, previous illnesses and individual immune responses, making standardised test interpretation difficult. Antibody detection indicates *Brucella* exposure and is not necessarily an active or recent infection [69, 71, 73, 76].

Several diagnostic assays have become obsolete over the years. The intradermal skin test, which cannot distinguish exposure levels, complicates later serodiagnostic tests by inducing antibody production. The opsonocytophagic index poses infection risks to laboratory workers and offers inconsistent results. Despite their use in some regions, haemagglutination tests lack global acceptance owing to these shortcomings [68].

#### 8.3.3. Molecular approaches and nucleic acid amplification tests (NAATs)

The genomic approach is accurate and rapid for detecting brucellosis in humans and animals, providing successful results even in ambiguous or asymptomatic cases. However, a positive result does not always signify an active infection, as it may detect genetic material from inactive or treated bacteria. Although sensitive nucleic acid amplification and serological tests are adequate for identifying brucellosis, culture remains the gold standard for its widespread clinical and epidemiological use. Peripheral blood is optimal for the molecular interpretation of human brucellosis. Other specimens from various systems can aid in diagnosing focal brucellosis, where cultures may be negative. Genetic materials from formalin-fixed, paraffin-embedded tissues can also be evaluated using established procedures.

Several gene targets have been used to diagnose *Brucella* infections. The 16S rRNA gene is a potential diagnostic target; however, there have been instances of cross-reactions that might lead to false-positive results. Thus, the IS711 insertion sequence is a potential target. However, its utility has been questioned because of sequence variations and absence in some *Brucella* strains, making it unreliable in certain contexts. Moreover, *bcsp31* is most frequently used for diagnosis as it encodes an immunogenic membrane protein. Its consistent presence and immune response make it the preferred choice for diagnostic testing [78–80]. Table 3 shows the amplification of various genes, including *omp2*, *omp31* and *bcsp31*, which have been targeted for molecular diagnosis; however, cross-reactions and variations in gene sequences may present challenges.

**Table 3.** Gene targets and primers are commonly utilised for the molecular detection of *Brucella* infection.

Gene target	Primer name	Primer sequence (5′–3′)	Product size (bp)	References
*omp2*	JPF	GCGCTCAGGCTGCCGACGCAA	193	[81]
	JPR	ACCAGCCATTGCGGTCGGTA		
*omp31*	F	TGGTAAGGTCAAGTCTGCGTT	281	[82]
	R	CTTCTTCATTCCGTGTTCGTG		
*omp28/bp26*	26A	GCCCCTGACATAACCCGCTT	1029	[83]
	26B	GAGCGTGACATTTGCCGATA		
*16S rRNA*	F4	TCGAGCGCCCGCAAG GGG	905	[80]
	R2	AACCATAGTGTCTCCACTAA		
*IS711*	I1	TCAATCCAACACGTTCC	52	[84]
	I2	TCCTTGTACAGCCTCC		
*bcsp31*	B4	TGGCTCGGTTGCCAATATCAA	223	[85]
	B5	CGCGCTTGCCTTTCAGGTCTG		

Real-time PCR assays that are species-specific and traditional *Brucella* ladder PCR assays are crucial for identifying and classifying *Brucella* species. The MLVA-16 (multilocus variable number tandem repeat analysis) panel, which targets 16 loci, is a reliable tool for diagnosing human brucellosis. These PCR-based methods provide specific and sensitive detection of *Brucella* and play a crucial role in confirming the presence of the pathogen and determining the species involved. Various amplification methodologies have been employed, including real-time PCR, multiplex PCR, nested PCR, PCR-enzyme immunoassay (PCR-EIA) and loop-mediated isothermal amplification (LAMP). Nested PCR involves using two sets of primers in two successive runs to boost the specificity and sensitivity of detection. The PCR-EIA was coupled with an enzyme immunoassay using a microplate setup to improve detection sensitivity. LAMP offers advantages such as simplicity, rapid response time and cost-effectiveness in limited resource settings.

Although genomic techniques are emerging, conventional methods, such as culture and serology assays, remain fundamental in diagnosing brucellosis and related infections by *Brucella* spp. Sending all *Brucella* strains to a reference laboratory for comprehensive species-level identification and biovar determination is essential for proper identification. This is vital for identifying the infection source, investigating outbreaks, tracking strains, differentiating isolates and assessing veterinary control strategies.

Traditional approaches to species differentiation, while reliable, are time-consuming, labour-intensive and present infection risks to lab workers. Molecular methods have emerged as quick and accurate alternatives. A fluorescence *in situ* hybridisation (FISH) test targeting the 16S rRNA gene permits the rapid and specific detection of human-pathogenic *Brucella* species. However, the limited polymorphism within the *Brucellaceae* family’s 16S rRNA gene makes it challenging to differentiate *Brucella* from closely related organisms, such as the *Ochrobactrum* genus.

Novel nucleic acid amplification methods have been developed to differentiate *between Brucella* species. These tests demonstrate high sensitivity and specificity. However, caution should be exercised when interpreting the NAAT results, as a positive test does not always indicate an ongoing infection. Instead, it could indicate a small number of bacteria present, DNA from non-viable organisms, or the presence of the pathogen in individuals who have already recovered from the infection [86]. Initially employed on peripheral blood with satisfactory results, serum samples are now considered the preferred choice for molecular detection of human brucellosis because of their higher effectiveness in yielding accurate diagnoses [87]. Furthermore, it is possible to use formalin-fixed paraffin-embedded (FFPE) tissue obtained from surgical biopsy samples for analysis if validated DNA extraction methods are employed [88].

Terrestrial *Brucella* species, including the strains used in vaccines, can be recognised and distinguished using the AMOS PCR test. Other PCR-based NAAT assays have been developed to identify specific *Brucella* and marine species rapidly. The Bruce Ladder multiplex PCR assay has high reproducibility and is species-specific. Additionally, five terrestrial *Brucella* species can be simultaneously detected using multiplex real-time PCR techniques based on single nucleotide polymorphisms (SNPs) [89].

##### 8.3.3.1. Advantages

Compared with bacteriological isolation, molecular methods offer several advantages regarding safety, sensitivity and speed. These methods enable rapid detection and differentiation of various bacterial species, particularly those with slow growth rates.

##### 8.3.3.2. Disadvantages

Comparative studies and standardised commercial molecular methods for brucellosis detection are limited, and the availability of next-generation sequencing technologies in low-income countries is insufficient. According to the OIE Terrestrial Manual (OIE 2016), no test can positively identify a bacterium such as *Brucella*. Integrating multiple techniques, including culture and serology, is necessary for the definitive diagnosis of *Brucella* infection [74].

#### 8.3.4. Important aspects regarding animal diagnosis

A definitive diagnosis of *Brucella* infection can be established through the isolation of the bacteria from tissue samples obtained during autopsy, milk or abortion. The most practical method for diagnosing *Brucella* infection is serology, which can be used to screen cattle using the Rose Bengal Test (RBT) and to confirm infection in specific animals using the enzyme-linked immunosorbent assay (ELISA) or Complement Fixation Test (CFT). For surveillance, milk samples can be screened using the milk ring test or ELISA. However, no serological test can confirm infection in specific animals such as sheep, goats and pigs. Serological testing should be applied on a herd or flock basis, and the skin test is useful for screening on a herd or flock level, particularly when immunisation is not used. For the diagnosis of *B. canis* infection, a ‘rough-specific’ antigen is required [90].

## 9. Current treatment methods for brucellosis

Doxycycline and rifampin are commonly used antibiotics for treating brucellosis. They form the basis for treating all types of human brucellosis. Following suitable antibiotic therapy, full recovery is expected in acute, uncomplicated brucellosis. Adults and children over eight usually take doxycycline, the preferred antibiotic due to its dosing frequency and fewer gastrointestinal side effects, orally for six weeks. To minimise the risk of relapse, aminoglycosides are often added during the initial 2–3 weeks of therapy [4, 35, 41, 91]. Although gentamicin shows promise, further research is needed to establish the optimal dosage and duration. Rifampicin is another effective alternative. A six-week oral administration of both doxycycline and rifampicin showed similar efficacy in treating uncomplicated brucellosis. Fluoroquinolones are considered secondary alternatives because of their high efficacy (Table 4).

**Table 4.** Recommendations for treating different cases of human brucellosis [4, 35, 41, 91].

Case	Treatment	
Uncomplicated brucellosis: adults and children ≥8 years	First line of treatment	Doxycycline 500 mg every six hours orally for 6 weeks or 2.2 mg/kg IV every 12 h
	Principal alternative therapy	Doxycycline (200 mg/day orally) + rifampicin (600–900 mg/day orally), with both drugs administered for 6 weeks. This regimen has generally been found to be of similar efficacy to doxycycline + streptomycin for patients with uncomplicated brucellosis
	Secondary alternative therapy	Fluoroquinolones, trimethoprim/sulfamethoxazole (TMP/SMZ, co-trimoxazole)
Children <8 years	Aminoglycosides, co-trimoxazole and rifampicin. TMP/SMZ (8/40 mg/kg/day twice daily orally) administered for 6 weeks + streptomycin (30 mg/kg/day once daily intramuscularly) administered for 3 weeks or gentamicin (5 mg/kg/day once daily intravenously or intramuscularly) administered for 7–10 days. Alternatives include TMP/SMZ + rifampicin (15 mg/kg/day orally), each administered for 6 weeks, or rifampicin + an aminoglycoside	
Complicated cases of brucellosis	Spondylitis	May require prolonged therapy, such as the continuation of doxycycline for 8 weeks or more
	Neurobrucellosis	Since tetracyclines and aminoglycosides do not penetrate the blood/brain barrier well, rifampicin or co-trimoxazole be added to the standard regimen of doxycycline + streptomycin for a minimum of 6–8 weeks
	*Brucella* endocarditis	Doxycycline + aminoglycoside rapidly kills the bacteria, as does rifampicin or co-trimoxazole. Prolonged therapy is recommended for at least 8 weeks
Pregnancy	Co-trimoxazole has been used in individual cases with reported success. Another alternative is rifampicin therapy for at least 45 days	
Post-exposure prophylaxis	Tetanus toxoid (when indicated) with doxycycline for 6 weeks	

While the WHO-recommended brucellosis treatment has evolved, the optimal approach remains unclear. This review suggests that a combination of doxycycline and aminoglycosides is common for uncomplicated brucellosis. Short-term treatment is discouraged because of the high failure and relapse rates. For complicated cases of spondylitis, neurobrucellosis or endocarditis, a prolonged triple therapy regimen involving streptomycin, gentamicin, doxycycline and rifampicin is more effective.

In resource-limited areas, various combinations of oral drugs such as tetracycline/rifampicin, doxycycline/ofloxacin or ciprofloxacin/rifampicin can be used. Rifampicin should be used cautiously and never alone to avoid multidrug-resistant tuberculosis. In children, co-trimoxazole combined with gentamicin or rifampicin is recommended, and quinolones should be used cautiously as a monotherapy.

For dogs with *B. suis* infection, a combination of rifampicin and doxycycline was administered. Euthanasia should be considered in severe cases to prevent zoonotic exposure. In *B. canis*-infected dogs, dual therapy is recommended despite the high relapse rates, particularly in males.

In production animals, brucellosis treatment is typically avoided, and the affected animals are usually culled. This varied information underscores the complexity of brucellosis treatment and different approaches, depending on the species affected [4, 35, 41, 91].

Antibiotic choices for brucellosis should consider factors such as patient details, drug availability and local resistance patterns. Close monitoring and follow-up are vital for successful treatment and prevention of relapse [91]. Despite this, treatment failure and relapse rates in mild brucellosis cases are common (5–15%), highlighting the need for continuous monitoring and repeated serological testing for one year.

The emergence of multidrug-resistant *Brucella* strains in endemic areas worldwide has been linked to improper antimicrobial use. The use of antibiotics in livestock contributes to this issue, posing a public health risk and limiting the availability of treatments. Regular antimicrobial susceptibility testing is essential for effective brucellosis management. Techniques such as microdilution, E-tests, Kirby Bauer and real-time PCR can help ascertain the minimum inhibitory concentrations (MICs) of drugs and assess the *Brucella* resistance profiles [35].

## 10. Antimicrobial resistance in *Brucella*

Challenges in managing the disease include the emergence of antibiotic-resistant strains and the need for continued treatment to avoid recurrence. The chronic nature of brucellosis, combined with the capacity of *Brucella* to reside within host cells and sequester at difficult-to-reach sites, can contribute to treatment relapse. The relapse rate in uncomplicated cases is estimated to be 5–15%. The cause of these relapses is unclear because of the emergence of antimicrobial resistance (AMR) or the inability to eradicate germs at the infection sites. However, studies on *Brucella* MICs in endemic regions have generally shown that bacteria remain susceptible to doxycycline and rifampicin, which are commonly used antibiotics for brucellosis treatment [65, 70, 92].

Several studies describing potential resistance to rifampin in brucellosis have been reported, for instance, from various countries throughout the globe. Rifampicin, co-trimoxazole (trimethoprim-sulfamethoxazole), ampicillin-sulbactam and colistin intermediate resistance phenotypes have also been reported (Table 5).

**Table 5.** Global antibiotic resistance pattern data.

Country	Resistance pattern	References
Norway	Resistant to rifampicin	[93]
Iran	Resistant to cotrimoxazole	[94]
Kazakhstan	48% resistance to rifampicin	[95]
China	Resistance to cotrimoxazole (7.0%) and rifampin (1.0%)	[96]
Peru	No resistance	[97]
UK	No resistance	[98]

The application of whole-genome sequencing (WGS) has enabled a more thorough identification of genes linked to virulence and resistance in *Brucella* strains. Even among strains recovered from various hosts, there was no discernible variation in AMR distribution and virulence genes between resistant and sensitive *B. abortus* and *B. melitensis* strains. Therefore, additional research on the antibiotic susceptibility of *Brucella* isolates is required. Although many microbes have benefited from research on resistance and virulence mechanisms at the genome level, they have limited value for *Brucella*. Future research should examine virulence mechanisms and resistance at proteomic and transcriptomic levels in *Brucella*.

## 11. β-Carbonic anhydrases (CAs) as potential targets

*Brucella* spp. are known to develop resistance to several clinically used drugs. Therefore, there is an urgent need to identify novel treatment strategies targeting unique bacterial pathways. *Brucella* genome sequencing has revealed potential drug targets, and metalloenzymes have emerged as promising candidates for novel treatments [99]. One such metalloprotein, histidine dehydrogenase (HDH), is vital for the intracellular growth of bacteria.

Further genomic studies identified two CAs (BR1829 and BRA0788 in *B. suis*) in *Brucella*. These enzymes, which are part of the β-class CA family and contain zinc as a metal ion, resemble β-CAs found in other pathogens [100, 101]. It has been demonstrated that the β-CAs of pathogenic microbes can be inhibited both *in vitro* and *in vivo* and, therefore, can be targeted using small molecular inhibitors. In living organisms, there are eight distinct classes of CAs: α, β, γ, δ, ζ, η, θ and ι [102]. Although humans only have α-CAs, pathogenic microbes can express multiple classes of CAs, including α, β and γ, which are present in both prokaryotic and eukaryotic organisms. Notably, most CAs are zinc-dependent and facilitate the conversion of carbon dioxide into bicarbonate and protons [103, 104].

β-CAs in *Brucella* play a role in several vital biosynthetic processes, some of which are critical for intracellular growth and virulence. These β-CAs are emerging as potential drug targets, offering a new avenue for developing antibacterial agents that do not share resistance patterns with existing antibiotics. Their significance is further underscored by their essential roles in the growth and virulence of various intracellular pathogens. This makes β-CAs a promising target for *Brucella* treatment without adverse side effects.

Recent studies have identified multiple inhibitors of β-CAs in pathogens, such as *Neisseria* spp., *H. pylori*, *B. suis*, *M. tuberculosis*, *S. pneumoniae* and pathogenic parasites, which effectively hamper their growth *in vitro* [100, 105–110] and *in vivo* [110, 111]. Detailed studies on *Brucella* β-CAs have revealed their susceptibility to inhibition by these compounds, from classical aromatic and heteroaromatic sulfonamides to carbohydrate-based entities. Specifically, β-CA1 in *Brucella* is sensitive to sulfamide, sulfamic acid, phenyl boronic/arsonic acid, and, to a lesser extent, diethyldithiocarbamate. β-CA2 shows pronounced inhibition by several anions [112].

Compounds such as acetazolamide, ethoxzolamide, topiramate and sulpiride have shown strong inhibitory effects against *Brucella* β-CAs *in vitro*. Specific sulfonamide-based carbonic anhydrase inhibitors (CAIs) have been demonstrated to stifle *Brucella* growth in cultures [113]. Evidence suggests that targeting *Brucella* β-CAs using CAIs may represent a promising strategy for combating brucellosis [112].

## 12. Vaccination as a strategy for controlling the spread of brucellosis

Vaccination is a prospective approach for controlling the spread of brucellosis, particularly in livestock. Various vaccines have been developed for animals, but their efficacies vary. However, no human vaccine is currently available.

Currently, there are no officially approved vaccines for brucellosis in humans. The absence of accessible vaccines hampers efforts to manage the disease in humans [22]. Consequently, controlling animal brucellosis is the most efficient approach to preventing human infection [114]. Since the early 1900s, investigations and scientific inquiries into creating vaccines for brucellosis have commenced. The development of brucellosis vaccines involves the development of inactivated, live-attenuated and rough-attenuated vaccines. Initially, inactivated vaccines were formulated as a preventive measure against the disease. However, they were later replaced with more immunologically potent live attenuated vaccines to control brucellosis [115]. However, current vaccines have certain drawbacks. For instance, some of these vaccines can potentially induce human infections and result in abortions in pregnant cattle.

Despite these limitations, they remain crucial for preventing and managing brucellosis and are used globally [5]. With advancements in molecular techniques, new vaccines based on genetic engineering have been developed. These innovative vaccines have replaced conventional vaccines to prevent and control brucellosis more effectively [116]. The following are the various types of brucellosis vaccines and their efficacies.

### 12.1. Live attenuated vaccines

Immunising animals effectively manages brucellosis, whereas human vaccines are not available. Live-attenuated vaccines, considered optimal for controlling animal brucellosis, have drawbacks, such as antibiotic resistance potential, diagnostic interference and residual virulence [117]. Widely used vaccines, such as *B. abortus* S19 and *B. melitensis* Rev1, exhibit these issues, complicating the differentiation between vaccinated and infected animals [118, 119]. The *Brucella* suis S2 vaccine exhibits a favourable immune response and cross-species protection [120] but has a limited range of host species [121].

Recent advances have focused on engineered live attenuated vaccines with deleted virulence genes that offer enhanced safety and immune responses [122]. For example, the *B. melitensis* 16M hfq mutant strain [123] and a mutant of the *B. melitensis* TcfSR promoter demonstrated significant protection and no interference with serodiagnostic tests [124]. Other potential vaccines, such as the M5-90ΔwboA mutant [118] and 6MΔwzt, have demonstrated reduced pathogenicity and improved defence mechanisms, although they exhibit sensitivity to polymyxin B [125].

The *2308DNodVDNodW* rough vaccine from the virulent *B. abortus* 2308 strain offers a significant immune response similar to the *B. ovis abcBA* (*BoabcBA*) vaccine [126] against the *B. melitensis* strain 16M [127]. Both ensure effective immunity and minimise diagnostic issues. Furthermore, the VTRS2 vaccine from *B. suis*, despite its sensitivity to polymyxin B [128] and detergents, and the rough mutant strain of *B. neotomae* show promising results in immune response and protection, showing the potential for further vaccine development in managing brucellosis [129].

### 12.2. Subunit vaccines

Creating effective vaccines for brucellosis poses a significant challenge because of highly virulent strains and specific tissue preferences. Subunit vaccines show potential in terms of safety, non-infectious and non-viable. However, their ability to mimic the replication of natural infections is limited [130]. Although subunit vaccines offer the benefit of being safe, they require multiple booster shots and the use of various antigens, adjuvants and delivery mechanisms to generate robust immunity and safeguard against brucellosis in cattle. However, this approach may not be economically feasible because of associated costs [131].

Additionally, it is crucial to recognise that the immune reactions observed in mice cannot precisely represent the immune reactions triggered in the hosts following vaccination. Therefore, further comprehensive research is required to discover recombinant vaccines incorporating multiple *Brucella* antigens. Unfortunately, despite numerous efforts, no effective subunit vaccine has been successfully developed for brucellosis [132]. Furthermore, multiple studies have demonstrated that subunit vaccines can generate protection and immune reactions comparable to those of attenuated vaccines [133–135]. However, it is essential to note that contradictory findings have been reported in other studies in which such equivalence was not observed [136].

### 12.3. Vaccines based on nanoparticles

In animal model experiments, nanoparticle-based oral vaccines incorporating the *Brucella* vaccine triggered antibody responses, including IgM, mucosal IgA and IgG. These vaccines have demonstrated notable advantages in animal studies, such as a more pronounced Th1–Th17 immune response [137]. However, due to the potential risk of disease transmission, nanoparticle-based vaccines cannot immunise humans against brucellosis [138]. The main drawbacks of these vaccines include toxicity, limitations in antigen loading and production, and suboptimal ability to stimulate the immune system [139]. The MAN-NP-HS vaccine candidate employs nanoparticle technology with mannosylation to target mannose receptors, thereby improving antigen uptake. This approach stimulates the production of mucosal IgA antibodies and Th1–Th2 cytokines, including IFN-γ, which promotes cellular immunity. Compared to Rev1, MAN-NP-HS provides better protection by inducing more specific IgA responses [140]. A promising vaccine candidate combines LPS and OPS antigens with PLGA nanoparticles. This approach aims to offer robust protection to both humans and animals by stimulating the production of IgM and IgG antibodies. Although adding these antigens without any combination is insufficient for inducing immune responses, their combination with nanoparticles increases antibody production [141].

### 12.4. DNA vaccines

DNA-based *Brucella* vaccines have demonstrated both safety and efficacy in combating brucellosis. These vaccines elicit robust cellular immune responses because of their ability to express antigens and incorporate CpG motifs. Additionally, DNA-based vaccines offer the advantage of simple storage conditions. They contain crucial gene sequences that play vital roles in the intracellular survival of *Brucella* spp. [142]. Extensive research has yielded compelling evidence for boosting immune responses and the effectiveness of diverse virulence genes in animal experiments. DNA vaccines have the potential to overcome the drawbacks associated with other brucellosis vaccines, as the vaccination of animals with various types of DNA vaccines has demonstrated complete immunisation to virulent strains [143]. DNA-based vaccines for brucellosis activate the immune system and promote the activation of TCD4 and TCD8 helper cells. These vaccines also lead to elevated levels of protective cytokines such as IFN-γ, TNF-α and IL-12, contributing to the immune response and defence against the disease [144].

Nevertheless, DNA-based vaccines do not confer substantial protection compared with live-attenuated vaccines. Research indicates no notable alterations in IL-4, IL-10 and IFN-γ expression, indicating an immune response to DNA-based vaccines [140]. The lack of significant protection offered by DNA-based vaccines could be attributed to their inability to effectively express specific antigens, such as the GroEL-Hsp antigen in the PcDNA3-DNA vaccine. Additionally, the need to repeatedly boost doses is associated with a diminished long-term immune response, which can be enhanced by incorporating an adjuvant into the vaccine formulation [145]. Although DNA-based vaccines express protective antigens, there may be limitations in the amount of antigen expression achieved. Ongoing efforts are being made to address this issue by developing strategies to prolong the expression of these genes and to prevent gene silencing over an extended period.

### 12.5. Vector vaccines

Live vector-based vaccines using *Brucella* as a delivery system have emerged as an effective method for delivering diverse antigens, whether heterologous or homologous. These genetically modified vaccines are formulated to trigger an antigen-specific T-cell immune response by replicating within host cells and producing multiple copies of the *Brucella* antigen [146]. Different viral and bacterial vectors, including *Salmonella*, *Escherichia coli*, *Salmonella* and Influenza viruses, can be used to express *Brucella* proteins. *Salmonella* offers advantages such as its inherent adjuvant effect, potential for single-dose vaccination, ability to present more than one antigen, and ability to penetrate natural barriers. However, it is essential to note that multiple *Salmonella* infections can lead to exacerbated disease outcomes in affected animals, potentially resulting in miscarriages and reduced productivity [147].

To address the absence of pre-existing immunity to the H5N1 influenza virus in humans, researchers have developed influenza viral vectors (IVVs) [148]. Lactic acid bacteria-based mucosal vaccines have demonstrated protective responses against various challenges. Nonetheless, these vaccines have the drawback of potentially disseminating genetically modified organisms with markers for drug resistance in both the host microbiota and the surrounding environment [149]. However, adenovirus-based vaccines have certain drawbacks, including substantial periods of temporary transgene expression, pre-existing immunity and high immunogenicity [150]. Several studies have assessed the potential of recombinant viral vector vaccines in formulating a potent human vaccine to combat human brucellosis infections. One study specifically examined an IVV of the H5N1 subtype that expressed *Brucella* Omp16, L7/L12, Omp19 and Cu-Zn SOD immunodominant proteins. The vaccine demonstrated significant protective effects when administered via intranasal and sublingual routes, comparable to the *B. melitensis* Rev1 vaccine [151].

Other studies have demonstrated that recombinant influenza A viruses of subtypes H5N1 and H1N1 can stimulate Th1 CD4+ and CD8+ T-cell immune responses, leading to effective protection against challenges [152]. Probiotics, such as *L. casei*, have been explored as vectors for strong immune responses and provide a high level of protection, similar to the IRIBA Strain Vac Calf vaccine. Releasing cytokines such as IFN-γ, IL-2 and IL-4 plays a crucial role in mediating cell-mediated immune responses. Mucosal vaccination using *L. casei* or *L. lactis* vector vaccines represents a potential vaccine delivery approach, with the advantage of reduced risk of eliciting immunological tolerance compared to persistent strains [153].

### 12.6. Recombinant peptides as a brucellosis vaccine

The use of recombinant peptides as brucellosis vaccines is a promising approach in the field of brucellosis prevention and control. Traditional vaccines like the Rev-1 vaccine have limitations, including the risk of abortion in pregnant animals and interference with diagnostic tests. In contrast, recombinant peptides offer safer and more targeted alternatives [154].

Research has demonstrated that recombinant peptides, such as rBtuB-Hia-FlgK, can stimulate specific immune responses, particularly Th1 and Th2 responses, which are crucial for combating *Brucella* infection. These peptides can promote the proliferation of CD4+ and CD8+ T cells and the production of key cytokines, including IFN-γ, TNF-α and IL-2, which play central roles in immune defence against *Brucella* [155].

One significant advantage of recombinant peptides is their ability to trigger an immune response similar to attenuated vaccines but without associated risks. This makes them a safer option for preventing brucellosis in livestock, such as goats, and potentially, in humans. Additionally, recombinant peptides can target specific *Brucella* species, enhancing vaccine specificity [155]. In conclusion, the development and use of recombinant peptides as brucellosis vaccines hold promise for overcoming the limitations of traditional vaccines, providing a safer and more effective means of preventing this zoonotic disease. Further research and development in this area could potentially lead to improved strategies for brucellosis control, benefiting both animal and human health.

## 13. Continued research and collaboration

Continued research and collaboration are essential for devising effective strategies to control and prevent brucellosis. The coordinated efforts of public health officials, healthcare providers and veterinary experts are vital to enhance diagnosis, treatment and prevention. They conduct thorough surveillance, monitoring and public awareness campaigns, emphasising the One Health approach, vaccination programs and biosecurity measures for livestock. During outbreaks, timely control measures such as animal quarantine and movement restrictions are crucial.

Healthcare providers play a significant role in diagnosing and treating infections early, implementing targeted screening and preventive measures, and educating patients about associated risks and prevention [156]. Veterinary experts focus on surveillance, early detection and implementation of animal disease control strategies. They also contribute to wildlife management efforts to minimise the brucellosis spillover between wildlife and livestock. Their active involvement in research, collaboration and the one-health approach plays a substantial role in brucellosis control and prevention [157].

Recommendations for controlling and preventing brucellosis include enhanced food safety regulations, hygiene practices and surveillance systems. Improved awareness and education for healthcare providers and the public are also imperative [158]. Assessing the current disease burden involves timely detection and obtaining precise data regarding potential carriers. Effective monitoring and control at the national level require collaboration between different governmental ministries and agencies [159].

The societal and economic impacts of zoonoses can be assessed using parameters such as disability-adjusted life years (DALY) for a comparative evaluation of the disease burden and for facilitating informed decision-making regarding brucellosis management programs [160, 161]. Historical instances of quarantine due to brucellosis, such as the exposure of British soldiers to *Brucella*-contaminated milk, highlight the importance of addressing the occurrence and transmission to eradicate the etiology through quarantine and elimination of infected animals [162].

Measures to reduce the risk of brucellosis transmission through milk and dairy products include comprehensive thermal cooking before consumption and enhancing the safety standards of dairy supply chains. Workers exposed to *Brucella*, such as veterinarians, laboratory workers and those handling infected animals, must be equipped with appropriate protective equipment and training [163].

Although no approved human vaccine targeting *Brucella* exists, the management of human brucellosis has relied on controlling animal brucellosis through vaccination. Despite some drawbacks, ongoing research on developing novel vaccines using innovative approaches, such as vector-based, recombinant and subunit vaccines, shows promise. Continued efforts in these areas could potentially aid in developing a human *Brucella* vaccine, further strengthening the fight against brucellosis.

## 14. Control and prevention of the spread of brucellosis

Controlling and preventing brucellosis involve enhancing food safety, hygiene and surveillance and increasing public and healthcare provider awareness. Despite efforts by the International Task Force for Disease Eradication in 1993, eradication has been hampered by inadequate facilities and resources. CDC follows the Dahlem Workshop guidelines for eradicating infectious diseases, focusing on thorough disease assessment and management [164].

Timely and accurate data on symptomatic or asymptomatic animal carriers are vital for assessing disease burden. Governmental collaboration aids in effective monitoring and control of outbreaks. Delays in disease reporting exacerbate this problem, increasing societal and economic impacts measured using the DALY parameter [165, 166].

Historical instances, such as the British services’ brucellosis quarantine in 1906, highlight the significance of a quick response in reducing the spread of the disease [167, 168]. Addressing both occurrence and transmission is of paramount importance. Despite the human role in disease spread among wildlife, international agreements, such as the Biological Weapons Convention of 1972, have helped in managing bioterrorism-related outbreaks [169].

Disease transmission mainly occurs through consuming raw or undercooked meat and unpasteurised dairy products, necessitating comprehensive preventive measures throughout the dairy and meat supply chain. Despite the resilience of *Brucella* species to various food-processing conditions, ensuring that all products undergo thorough cooking before consumption is vital [6, 170].

The WHO classifies *Brucella* in risk group 3, highlighting the significant risk for individuals such as veterinarians, laboratory workers and butchers, who frequently handle these bacteria and underscore the need for proper protective equipment and training [3, 6].

Currently, there is no FDA-approved human vaccine for *Brucella*, although China uses two live-attenuated vaccines targeting *Brucella melitensis* and *Brucella suis* strains, with limited international approval [6, 171]. Control of human brucellosis predominantly relies on animal vaccination [114, 172]. Despite its effectiveness, concerns over vaccination include potential abortion in animals, virulence towards humans, and the emergence of antibiotic-resistant strains. Ongoing research exploring novel vaccines using advanced methods holds promise for more effective and globally accepted *Brucella* vaccines. These vaccines use cutting-edge approaches such as reverse vaccinology, novel additives, structural vaccinology and generalised modules for membrane antigens (GMMA) [173]. These approaches can potentially shed light on developing a human *Brucella* vaccine.

## 15. Conclusions

In conclusion, understanding the biological aspects of a disease is pivotal for its effective management, including tailored therapies and early detection. While ongoing research on disease mechanisms informs vaccine development, the extensive time required underscores the importance of continued drug discovery. Rapid growth in multi-omics and bioinformatics has significantly aided patient profiling and potential drug targeting, bolstering novel drugs and vaccine development [172, 174].

The cross-sector collaboration marks a significant step towards a comprehensive control program, necessitating community-wide active participation and endorsement. A multidisciplinary approach allows for transparent data exchange and implementation of an empirical surveillance model for accurate brucellosis tracking [175]. Addressing the gap between socioeconomic challenges and research priorities involves prioritising funding for infrastructure and human resources. International collaboration is vital, as seen in the World Bank’s recent $82 million grant to India for zoonosis and endemic disease prevention [176]. This collective effort is essential for effectively managing and controlling zoonoses globally.

## Data Availability

Data sharing is not applicable to this article as no new data were created in this study.

## References

[1] Pappas G, Papadimitriou P, Akritidis N, et al. The new global map of human brucellosis. Lancet Infect Dis. 2006;6(2):1–24. doi: 10.1016/S1473-3099(06)70382-6. 16439329

[2] Atluri VL, Xavier MN, De Jong MF, et al. Interactions of the human pathogenic *Brucella* species with their hosts. Annu Rev Microbiol. 2011;65(1):523–541. doi: 10.1146/annurev-micro-090110-102905. 21939378 PMC13363517

[3] Franco MP, Mulder M, Gilman RH, et al. Human brucellosis. Lancet Infect Dis. 2007;7(12):775–786. doi: 10.1016/S1473-3099(07)70286-4. 18045560

[4] Ariza J, Bosilkovski M, Cascio A, et al. Perspectives for the treatment of brucellosis in the 21st century: the Ioannina recommendations. PLoS Med. 2007;4(12):e317. doi: 10.1371/journal.pmed.0040317. 18162038 PMC2222927

[5] Schurig GG, Sriranganathan N, Corbel MJ. Brucellosis vaccines: past, present and future. Vet Microbiol. 2002;90(1–4):479–496. doi: 10.1016/S0378-1135(02)00255-9. 12414166

[6] Goonaratna C. Brucellosis in humans and animals. Ceylon Med J. 2009;52(2):66. doi: 10.4038/cmj.v52i2.1028.

[7] Bennett NJ. *Brucellosis*. Medscape; 2023. Available from: https://emedicine.medscape.com/article/213430-overview?form=fpf

[8] Brucellosis; 2023 [cited 2023 Dec 8]. Available from: https://www.who.int/news-room/fact-sheets/detail/brucellosis

[9] Centers for Disease Control and Prevention. Estimates human Brucella infections could be four times higher than previously thought. Food Safety; 2023. Available from: https://www.food-safety.com/articles/8817-cdc-estimates-human-brucella-infections-could-be-four-times-higher-than-previously-thought.

[10] Laine CG, Johnson VE, Scott HM, et al. Global estimate of human brucellosis incidence. Emerg Infect Dis. 2023;29(9):1789–1797. doi: 10.3201/eid2909.230052. 37610167 PMC10461652

[11] Khurana SK, Sehrawat A, Tiwari R, et al. Bovine brucellosis–a comprehensive review. Vet Q. 2021;41(1):61–88. doi: 10.1080/01652176.2020.1868616. 33353489 PMC7833053

[12] Pal M, Gizaw F, Fekadu G, et al. Public health and economic importance of bovine brucellosis: an overview. Am J Epid Inf Dis. 2017;5(2):27–34. doi: 10.12691/ajeid-5-2-2.

[13] Bano Y, Ahmad Lone S. Brucellosis: an economically important infection. J Med Microb Diagn. 2015;4(4):208. doi: 10.4172/2161-0703.1000208.

[14] Fritz CL, Nguyen A, Vugia DJ. Epidemiology of brucellosis in California, 1993–2017: a continuing foodborne disease risk for older Latinos. Clin Infect Dis. 2021;73(11):2023–2030. doi: 10.1093/cid/ciab551. 34134141

[15] Uzunović S, Skomorac M, Bašić F, et al. Human brucellosis as an epidemic zoonosis in Zenica-Doboj canton (Bosnia and Herzegovina) during 2008–2018. Open Infect Dis J. 2020;12(1):1–6. doi: 10.2174/1874279302012010001.

[16] Djokic V, Freddi L, de Massis F, et al. The emergence of *Brucella canis* as a public health threat in Europe: what we know and what we need to learn. Emerg Microb Infect. 2023;12(2):2249126. doi: 10.1080/22221751.2023.2249126. PMC10540651 37649455

[17] Ali S, Nawaz Z, Akhtar A, et al. Epidemiological investigation of human brucellosis in Pakistan. Jundishapur J Microbiol. 2018;11(7):e61764. doi: 10.5812/jjm.61764.

[18] Kalin R, Karahan M, Acik MN, et al. Mastitisli inek sütlerinde önemli patojenlerin direkt tespiti için bir multipleks PCR yönteminin geliştirilmesi. Kafkas Univ Vet Fak Derg. 2017;23(6):925–931. doi: 10.9775/kvfd.2017.17995.

[19] Yumuk Z, O’Callaghan D. Brucellosis in Turkey—an overview. Int J Infect Dis. 2012;16(4):e228–e235. doi: 10.1016/j.ijid.2011.12.011. 22333223

[20] Alavi SM, Motlagh ME. A review of epidemiology, diagnosis and management of brucellosis for general physicians working in the Iranian Health Network. Jundishapur J Microbiol. 2012;5(2):384–387. doi: 10.5812/jjm.3248.

[21] Bagheri H, Tapak L, Karami M, et al. Epidemiological features of human brucellosis in Iran (2011–2018) and prediction of brucellosis with data-mining models. J Res Health Sci. 2019;19(4):e00462. https://www.ncbi.nlm.nih.gov/pmc/articles/PMC7183567/32291361 PMC7183567

[22] Etemadi A, Moniri R, Saffari M, et al. Epidemiological, molecular characterization and risk factors of human brucellosis in Iran. Asian Pac J Trop Med. 2020;13(4):169. doi: 10.4103/1995-7645.280224.

[23] Nematollahi S, Ayubi E, Karami M, et al. Epidemiological characteristics of human brucellosis in Hamadan province during 2009–2015: results from the national notifiable diseases surveillance system. Int J Infect Dis. 2017;61:56–61. doi: 10.1016/j.ijid.2017.06.002. 28602765

[24] Norouzinezhad F, Erfani H, Norouzinejad A, et al. Epidemiological characteristics and trend in the incidence of human brucellosis in Iran from 2009 to 2017. J Res Health Sci. 2021;21(4):e00535. doi: 10.34172/jrhs.2021.70. 36511231 PMC8957668

[25] Al-Amr M, Abasi L, Khasawneh R, et al. Epidemiology of human brucellosis in military hospitals in Jordan: a five-year study. J Infect Dev Ctries. 2022;16(12):1870–1876. doi: 10.3855/jidc.16861. 36753655

[26] Holt HR, Bedi JS, Kaur P, et al. Epidemiology of brucellosis in cattle and dairy farmers of rural Ludhiana, Punjab. PLoS Negl Trop Dis. 2021;15(3):e0009102. doi: 10.1371/journal.pntd.0009102. 33735243 PMC8034737

[27] Nawaz Z, Shafique M, Zahoor MA, et al. Sero-epidemiology and risk factor analysis of human brucellosis in Punjab, Pakistan: a cross sectional study. Trop Biomed. 2021;38(3):413–419. doi: 10.47665/tb.38.3.084. 34608115

[28] Shi Y, Gao H, Pappas G, et al. Clinical features of 2041 human brucellosis cases in China. PLOS One. 2018;13(11):e0205500. doi: 10.1371/journal.pone.0205500. 30476930 PMC6258468

[29] Tao Z, Chen Q, Chen Y, et al. Epidemiological characteristics of human brucellosis—China. China CDC Wkly. 2021;3(6):114–119. doi: 10.46234/ccdcw2021.030. 34595016 PMC8393115

[30] Liu Z G, Wang M, Ta N, et al. Seroprevalence of human brucellosis and molecular characteristics of *Brucella* strains in Inner Mongolia autonomous region of China, from 2012 to 2016. Emerg Microbes Infect. 2020;9(1):263–274. doi: 10.1080/22221751.2020.1720528. 31997725 PMC7034055

[31] Djangwani J, Ooko Abong’ G, Gicuku Njue L, et al. Brucellosis: prevalence with reference to East African community countries–a rapid review. Vet Med Sci. 2021;7(3):851–867. doi: 10.1002/vms3.425. 33421354 PMC8136958

[32] Vigeant P, Mendelson J, Miller MA. Human to human transmission of *Brucella melitensis*. Can J Infect Dis. 1995;6(3):153–155. https://www.ncbi.nlm.nih.gov/pmc/articles/PMC3327908/ doi: 10.1155/1995/909404. 22514390 PMC3327908

[33] Shakir R. Brucellosis. J Neurol Sci. 2021;420:117280. doi: 10.1016/j.jns.2020.117280. 33358192

[34] Delam H, Keshtkaran Z, Rezaei B, et al. Changing patterns in epidemiology of brucellosis in the South of Iran (2015–2020): based on Cochran–Armitage trend test. Ann Glob Health. 2022;88(1):11. doi: 10.5334/aogh.3474. 35223430 PMC8833262

[35] Centers for Disease Control and Prevention. Treatment. Brucellosis. CDC; 2012. Available from: https://www.cdc.gov/brucellosis/treatment/index.html

[36] De Figueiredo P, Ficht TA, Rice-Ficht A, et al. Pathogenesis and immunobiology of brucellosis: review of *Brucella*–host interactions. Am J Pathol. 2015;185(6):1505–1517. doi: 10.1016/j.ajpath.2015.03.003. 25892682 PMC4450313

[37] Barbuddhe SB, Vergis J, Rawool DB. Immunodetection of bacteria causing brucellosis. In: Gurtler V, Patrauchan M, editors. Methods in microbiology. Vol. 47. Academic Press; 2020. p. 75–115. doi: 10.1016/bs.mim.2019.11.003.

[38] Matle I, Ledwaba B, Madiba K, et al. Characterisation of *Brucella* species and biovars in South Africa between 2008 and 2018 using laboratory diagnostic data. Vet Med Sci. 2021;7(4):1245–1253. doi: 10.1002/vms3.483. 33974356 PMC8294379

[39] Byndloss MX, Tsolis RM. *Brucella* spp. virulence factors and immunity. Annu Rev Anim Biosci. 2016;4(1):111–127. doi: 10.1146/annurev-animal-021815-111326. 26734887

[40] Elrashedy A, Gaafar M, Mousa W, et al. Immune response and recent advances in diagnosis and control of brucellosis. Ger J Vet Res. 2022;2(1):10–24. doi: 10.51585/gjvr.2022.1.0033.

[41] Głowacka P, Żakowska D, Naylor K, et al. *Brucella*–virulence factors, pathogenesis and treatment. Pol J Microbiol. 2018;67(2):151–161. doi: 10.21307/pjm-2018-029. 30015453 PMC7256693

[42] Wareth G, Melzer F, Neubauer H. In *Brucella*: selective pressure may turn some genes on instead of default off position. Med Hypotheses. 2017;103:29–31. doi: 10.1016/j.mehy.2017.04.006. 28571803

[43] Ulu Kilic A, Metan G, Alp E. Clinical presentations and diagnosis of brucellosis. Recent Pat Antiinfect Drug Discov. 2013;8(1):34–41. doi: 10.2174/1574891X11308010007. 22873352

[44] Centers for Disease Control and Prevention (CDC). Brucellosis reference guide: exposures, testing, and prevention. Atlanta (GA): The Center for Food Security and Public Health; 2018. p. 1–40.

[45] Young EJ. An overview of human brucellosis. Clin Infect Dis. 1995;21(2):283–289; quiz 290. doi: 10.1093/clinids/21.2.283. 8562733

[46] Rubach MP, Halliday JEB, Cleaveland S, et al. Brucellosis in low-income and middle-income countries. Curr Opin Infect Dis. 2013;26(5):404–412. doi: 10.1097/QCO.0b013e3283638104. 23963260 PMC3888775

[47] Heavey E. Brucellosis: a global concern. Nursing. 2019;49(5):14–16. doi: 10.1097/01.NURSE.0000554623.05347.a0. 31022022

[48] Çavuş B, Çaydaşı Ö, Aktan A, et al. Brucellosis as the cause of non-viral bacterial hepatitis: a case report. Open Access Maced J Med Sci. 2018;6(7):1260–1262. doi: 10.3889/oamjms.2018.198. 30087732 PMC6062275

[49] Güçlü M, Yakar T, Habeoğlu MA. Spontaneous bacterial peritonitis and chylothorax related to *Brucella* infection in a cirrhotic patient. Electron J Gen Med. 2007;4(4):201–204. doi: 10.29333/ejgm/82530.

[50] Makaritsis KP, Liaskos C, Papadamou G, et al. Spontaneous bacterial peritonitis: an unusual manifestation of brucellosis in a previous healthy male patient. BMJ Case Rep. 2015;2015(1):bcr2015209387. doi: 10.1136/bcr-2015-209387. PMC4420813 25903208

[51] Qiu Z, Yang F, Zhang S. Immune thrombocytopenic purpura and its rare association with a *Brucella* infection: a case report. Cureus. 2022;14(10):e30049. doi: 10.7759/cureus.30049. 36381884 PMC9637444

[52] Yamashita H, Takahashi Y, Kaneko H, et al. Thrombotic thrombocytopenic purpura with an autoantibody to ADAMTS13 complicating Sjögren’s syndrome: two cases and a literature review. Mod Rheumatol. 2013;23(2):365–373. doi: 10.3109/s10165-012-0644-7. 22526830

[53] Lambourne JR, Brooks T. *Brucella* and *Coxiella*; if you don’t look, you don’t find. Clin Med. 2015;15(2):212. doi: 10.7861/clinmedicine.15-2-212. PMC4954535 25650208

[54] Li Q, Liu J, Jiang W, et al. A case of brucellosis-induced Guillain–Barre syndrome. BMC Infect Dis. 2022;22(1):72. doi: 10.1186/s12879-021-07025-3. 35057735 PMC8781241

[55] Essrani R, Shnitser A. *Brucella melitensis*-induced transaminitis. Cureus. 2021;13(3):e13656. doi: 10.7759/cureus.13656. 33824807 PMC8012176

[56] Young EJ. Clinical manifestations of human brucellosis. In: Young EJ and Corbel MJ, editors. Brucellosis: clinical and laboratory aspects. CRC Press; 2020. p. 97–126. doi: 10.1201/9781003068518-10.

[57] Kaya F, Kocyigit A, Kaya C, et al. Brucellar testicular abscess presenting as a testicular mass: can color Doppler sonography be used in differentiation? Turk J Emerg Med. 2015;15(1):43–46. doi: 10.5505/1304.7361.2014.82698. 27331193 PMC4909939

[58] Raju IT, Solanki R, Patnaik AN, et al. *Brucella endocarditis*—a series of five case reports. Indian Heart J. 2013;65(1):72–77. doi: 10.1016/j.ihj.2012.12.017. 23438616 PMC3860836

[59] Bazzazi N, Yavarikia A, Keramat F. Ocular involvement of brucellosis. Middle East Afr J Ophthalmol. 2013;20(1):95–97. doi: 10.4103/0974-9233.106407. 23580863 PMC3617540

[60] Karaali Z, Baysal B, Poturoglu S, et al. Cutaneous manifestations in brucellosis. Indian J Dermatol. 2011;56(3):339–340. doi: 10.4103/0019-5154.82505. 21772606 PMC3132922

[61] Korkmaz P, Doyuk Kartal E. Skin manifestations associated with brucellosis. EMJ Dermatol. 2016;4:119–125. doi: 10.33590/emjdermatol/10312753.

[62] Wang W, Lu X, Li C, et al. A man with recurrent fever, arthritis, and rashes—brucellosis? A case report. BMC Infect Dis. 2020;20(1):18. doi: 10.1186/s12879-019-4746-0. 31910802 PMC6947870

[63] Spink WW. What is chronic brucellosis? Ann Intern Med. 1951;35(2):358–374. doi: 10.7326/0003-4819-35-2-358. 14857606

[64] Ariza J, Corredoira J, Pallares R, et al. Characteristics of and risk factors for relapse of brucellosis in humans. Clin Infect Dis. 1995;20(5):1241–1249. doi: 10.1093/clinids/20.5.1241. 7620005

[65] Solera J. Update on brucellosis: therapeutic challenges. Int J Antimicrob Agents. 2010;36(Suppl. 1):S18–S20. doi: 10.1016/j.ijantimicag.2010.06.015. 20692127

[66] Akkoc G, Tekerek S. Osteoarticular involvement in childhood brucellosis: evaluation of clinical, laboratory and radiologic features of 185 cases. Pediatr Infect Dis J. 2023;42(5):381–388. doi: 10.1097/INF.0000000000003844. 36795549

[67] Whatmore AM, Koylass MS, Muchowski J, et al. Extended multilocus sequence analysis to describe the global population structure of the genus *Brucella*: phylogeography and relationship to biovars. Front Microbiol. 2016;7:2049. doi: 10.3389/fmicb.2016.02049. 28066370 PMC5174110

[68] Pappas G, Akritidis N, Bosilkovski M, et al. Medical progress brucellosis. N Engl J Med. 2005;352(22):2325–2336. doi: 10.1056/NEJMra050570. 15930423

[69] Yagupsky P, Morat P, Colmenero JD. Laboratory diagnosis of human brucellosis. Clin Microbiol Rev. 2020;33(1):e00073-19. doi: 10.1128/CMR.00073-19. PMC6860005 31722888

[70] Pappas G, Papadimitriou P. Challenges in *Brucella bacteraemia*. Int J Antimicrob Agents. 2007;30(Suppl. 1):29–31. doi: 10.1016/j.ijantimicag.2007.06.011. 17706402

[71] Olitzky AL. Laboratory techniques in brucellosis. Harefuah. 1976;91(11):408–409. 1017740

[72] Castaneda MR. A practical method for routine blood cultures in brucellosis. Proc Soc Exp Biol Med. 1947;64(1):114–115. doi: 10.3181/00379727-64-15717. 20285481

[73] Al-Attas RA, Al-Khalifa M, Al-Qurashi AR, et al. Evaluation of PCR, culture and serology for the diagnosis of acute human brucellosis. Ann Saudi Med. 2000;20(3–4):224–228. doi: 10.5144/0256-4947.2000.224. 17322662

[74] OIE-WOAH. Terrestrial code online access - WOAH - World organisation for animal health. 2023. https://www.woah.org/en/what-we-do/standards/codes-and-manuals/terrestrial-code-online-access/.

[75] Wareth G, Pletz MW, Neubauer H, et al. Proteomics of *Brucella*: technologies and their applications for basic research and medical microbiology. Microorganisms. 2020;8(5):766. doi: 10.3390/microorganisms8050766. 32443785 PMC7285364

[76] Di Bonaventura G, Angeletti S, Ianni A, et al. Microbiological laboratory diagnosis of human brucellosis: an overview. Pathogens. 2021;10(12):1623. doi: 10.3390/pathogens10121623. 34959578 PMC8709366

[77] El-Diasty M, El-Said R, Abdelkhalek A. Seroprevalence and molecular diagnosis of sheep brucellosis in Dakahlia Governorate, Egypt. Ger J Vet Res. 2021;1(1):34–39. doi: 10.51585/gjvr.2021.0006.

[78] Baddour MM, Alkhalifa DH. Evaluation of three polymerase chain reaction techniques for detection of *Brucella* DNA in peripheral human blood. Can J Microbiol. 2008;54(5):352–357. doi: 10.1139/W08-017. 18449219

[79] Bounaadja L, Albert D, Chénais B, et al. Real-time PCR for identification of *Brucella* spp.: a comparative study of IS711, bcsp31 and per target genes. Vet Microbiol. 2009;137(1–2):156–164. doi: 10.1016/j.vetmic.2008.12.023. 19200666

[80] Romero C, Gamazo C, Pardo M, et al. Specific detection of *Brucella* DNA by PCR. J Clin Microbiol. 1995;33(3):615–617. doi: 10.1128/jcm.33.3.615-617.1995. 7538508 PMC227999

[81] Leal-Klevezas DS, Martinez-Vazquez IO, Lopez-Merino A, et al. Single-step PCR for detection of *Brucella* spp. from blood and milk of infected animals. J Clin Microbiol. 1995;33(12):3087–3090. doi: 10.1128/jcm.33.12.3087-3090.1995. 8586678 PMC228649

[82] Kattar MM, Zalloua PA, Araj GF, et al. Development and evaluation of real-time polymerase chain reaction assays on whole blood and paraffin-embedded tissues for rapid diagnosis of human brucellosis. Diagn Microbiol Infect Dis. 2007;59(1):23–32. doi: 10.1016/j.diagmicrobio.2007.04.002. 17532591

[83] Mitka S, Anetakis C, Souliou E, et al. Evaluation of different PCR assays for early detection of acute and relapsing brucellosis in humans in comparison with conventional methods. J Clin Microbiol. 2007;45(4):1211–1218. doi: 10.1128/JCM.00010-06. 17267626 PMC1865811

[84] Al Nakkas AF, Wright SG, Mustafa AS, et al. Single-tube, nested PCR for the diagnosis of human brucellosis in Kuwait. Ann Trop Med Parasitol. 2002;96(4):397–403. doi: 10.1179/000349802125001203. 12171621

[85] Baily GG, Krahn JB, Drasar BS, et al. Detection of *Brucella melitensis* and *Brucella abortus* by DNA amplification. J Trop Med Hyg. 1992;95(4):271–275. 1495123

[86] Navarro E, Escribano J, Fernández J, et al. Comparison of three different PCR methods for detection of *Brucella* spp. in human blood samples. FEMS Immunol Med Microbiol. 2002;34(2):147–151. doi: 10.1111/j.1574-695x.2002.tb00616.x. 12381466

[87] Zerva L, Bourantas K, Mitka S, et al. Serum is the preferred clinical specimen for diagnosis of human brucellosis by PCR. J Clin Microbiol. 2001;39(4):1661–1664. doi: 10.1128/JCM.39.4.1661-1664.2001. 11283112 PMC87995

[88] Li M, Zhou X, Li J, et al. Real-time PCR assays for diagnosing brucellar spondylitis using formalin-fixed paraffin-embedded tissues. Medicine. 2018;97(9):e0062. doi: 10.1097/MD.0000000000010062. 29489665 PMC5851713

[89] Gopaul KK, Sells J, Lee R, et al. Development and assessment of multiplex high resolution melting assay as a tool for rapid single-tube identification of five *Brucella* species. BMC Res Notes. 2014;7(1):903. doi: 10.1186/1756-0500-7-903. 25495428 PMC4307374

[90] Rajni Joshi NS. Clinical, serological and molecular diagnosis of brucellosis in domestic animals. Int J Curr Microbiol Appl Sci. 2021;10(4):368–385. doi: 10.20546/ijcmas.2021.1004.040.

[91] Bosilkovski M, Keramat F, Arapović J. The current therapeutical strategies in human brucellosis. Infection. 2021;49(5):823–832. doi: 10.1007/s15010-021-01586-w. 33650077

[92] Patra S, Kalwaje Eshwara V, Pai AR, et al. Evaluation of clinical, diagnostic features and therapeutic outcome of neurobrucellosis: a case series and review of literature. Int J Neurosci. 2022;132(11):1080–1090. doi: 10.1080/00207454.2020.1860969. 33287603

[93] Johansen TB, Scheffer L, Jensen VK, et al. Whole-genome sequencing and antimicrobial resistance in *Brucella melitensis* from a Norwegian perspective. Sci Rep. 2018;8(1):8538. doi: 10.1038/s41598-018-26906-3. 29867163 PMC5986768

[94] Reza Irajian G, Masjedian Jazi F, Mirnejad R, et al. Species-specific PCR for the diagnosis and determination of antibiotic susceptibilities of *Brucella* strains isolated from Tehran, Iran. Iran J Pathol. 2016;11(3):238–247. 27799972 PMC5079456

[95] Shevtsov A, Syzdykov M, Kuznetsov A, et al. Antimicrobial susceptibility of *Brucella melitensis* in Kazakhstan. Antimicrob Resist Infect Control. 2017;6(1):130. doi: 10.1186/s13756-017-0293-x. 29299304 PMC5745643

[96] Doimari S, Singh V, Kumari R, et al. In vitro antimicrobial susceptibility of *Brucella* species isolated from human and animals in India. J Antibiot Res. 2019;3:1–6.

[97] Maves RC, Castillo R, Guillen A, et al. Antimicrobial susceptibility of *Brucella melitensis* isolates in Peru. Antimicrob Agents Chemother. 2011;55(3):1279–1281. doi: 10.1128/AAC.00979-10. 21199926 PMC3067062

[98] Cooke RPD, Perrett L. Antimicrobial susceptibility data for *Brucella melitensis* isolates cultured from UK patients. J Infect. 2014;68(4):401. doi: 10.1016/j.jinf.2014.01.002. 24440739

[99] Supuran CT. Carbonic anhydrases: novel therapeutic applications for inhibitors and activators. Nat Rev Drug Discov. 2008;7(2):168–181. doi: 10.1038/nrd2467. 18167490

[100] Aspatwar A, Kairys V, Rala S, et al. *Mycobacterium tuberculosis* β-carbonic anhydrases: novel targets for developing antituberculosis drugs. Int J Mol Sci. 2019;20(20):5153. doi: 10.3390/ijms20205153. 31627429 PMC6834203

[101] Köhler S, Ouahrani-Bettache S, Winum JY. *Brucella suis* carbonic anhydrases and their inhibitors: towards alternative antibiotics? J Enzym Inhib Med Chem. 2017;32(1):683–687. doi: 10.1080/14756366.2017.1295451. PMC6009918 28274160

[102] Aspatwar A, Tolvanen MEE, Barker H, et al. Carbonic anhydrases in metazoan model organisms: molecules, mechanisms, and physiology. Physiol Rev. 2022;102(3):1327–1383. doi: 10.1152/physrev.00018.2021. 35166161

[103] Aspatwar A, Parvathaneni NK, Barker H, et al. Design, synthesis, in vitro inhibition and toxicological evaluation of human carbonic anhydrases I, II and IX inhibitors in 5-nitroimidazole series. J Enzyme Inhib Med Chem. 2020;35(1):109–117. doi: 10.1080/14756366.2019.1685510. 31687859 PMC6844379

[104] Aspatwar A, Supuran CT, Waheed A, et al. Mitochondrial carbonic anhydrase VA and VB: properties and roles in health and disease. J Physiol. 2023;601(2):257–274. doi: 10.1113/JP283579. 36464834 PMC10107955

[105] Abdoli M, Bonardi A, Paoletti N, et al. Inhibition studies on human and mycobacterial carbonic anhydrases with N-((4-sulfamoylphenyl)carbamothioyl) amides. Molecules. 2023;28(10):4020. doi: 10.3390/molecules28104020. 37241761 PMC10222120

[106] Aspatwar A, Barker H, Aisala H, et al. Cloning, purification, kinetic and anion inhibition studies of a recombinant β-carbonic anhydrase from the Atlantic salmon parasite platyhelminth *Gyrodactylus salaris*. J Enzyme Inhib Med Chem. 2022;37(1):1577–1586. doi: 10.1080/14756366.2022.2080818. 35637617 PMC9176631

[107] Aspatwar A, Bonardi A, Aisala H, et al. Sulphonamide inhibition studies of the β-carbonic anhydrase GsaCAβ present in the salmon platyhelminth parasite *Gyrodactylus salaris*. J Enzyme Inhib Med Chem. 2023;38(1):2167988. doi: 10.1080/14756366.2023.2167988. 36647786 PMC9848252

[108] Aspatwar A, Hammaren M, Parikka M, et al. In vitro inhibition of *Mycobacterium tuberculosis* β-carbonic anhydrase 3 with mono- and dithiocarbamates and evaluation of their toxicity using zebrafish developing embryos. J Enzyme Inhib Med Chem. 2020;35(1):65–71. doi: 10.1080/14756366.2019.1683007. 31663386 PMC6830242

[109] Aspatwar A, Winum JY, Carta F, et al. Carbonic anhydrase inhibitors as novel drugs against mycobacterial β-carbonic anhydrases: an update on in vitro and in vivo studies. Molecules. 2018;23(11):2911. doi: 10.3390/molecules23112911. 30413024 PMC6278287

[110] Supuran CT. Bacterial carbonic anhydrases as drug targets: toward novel antibiotics? Front Pharmacol. 2011;2:34. doi: 10.3389/fphar.2011.00034. 21779249 PMC3132667

[111] Aspatwar A, Hammarén M, Koskinen S, et al. β-CA-specific inhibitor dithiocarbamate Fc14–584B: a novel antimycobacterial agent with potential to treat drug-resistant tuberculosis. J Enzyme Inhib Med Chem. 2017;32(1):832–840. doi: 10.1080/14756366.2017.1332056. 28629306 PMC6445161

[112] Maresca A, Scozzafava A, Köhler S, et al. Inhibition of beta-carbonic anhydrases from the bacterial pathogen *Brucella suis* with inorganic anions. J Inorg Biochem. 2012;110:36–39. doi: 10.1016/j.jinorgbio.2012.02.009. 22459172

[113] Vullo D, Nishimori I, Scozzafava A, et al. Inhibition studies of a β-carbonic anhydrase from *Brucella suis* with a series of water soluble glycosyl sulfanilamides. Bioorg Med Chem Lett. 2010;20(7):2178–2182. doi: 10.1016/j.bmcl.2010.02.042. 20211561

[114] Heidary M, Dashtbin S, Ghanavati R, et al. Evaluation of brucellosis vaccines: a comprehensive review. Front Vet Sci. 2022;9:925773. doi: 10.3389/fvets.2022.925773. 35923818 PMC9339783

[115] Montaraz JA, Winter AJ. Comparison of living and nonliving vaccines for *Brucella abortus* in BALB/c mice. Infect Immun. 1986;53(2):245–251. doi: 10.1128/iai.53.2.245-251.1986. 3089933 PMC260865

[116] Yang X, Skyberg JA, Cao L, et al. Progress in *Brucella* vaccine development. Front Biol. 2013;8(1):60–77. doi: 10.1007/s11515-012-1196-0. PMC3666581 23730309

[117] Mansoori N, Pourmand MR. Vaccines and vaccine candidates against brucellosis. Infect Epidemiol Microbiol. 2016;2(4):32–36. doi: 10.18869/modares.iem.2.4.32.

[118] Li ZQ, Shi JX, Fu WD, et al. A *Brucella melitensis* M5-90 wboA deletion strain is attenuated and enhances vaccine efficacy. Mol Immunol. 2015a;66(2):276–283. doi: 10.1016/j.molimm.2015.04.004. 25899866

[119] Truong QL, Cho Y, Kim K, et al. Booster vaccination with safe, modified, live-attenuated mutants of *Brucella abortus* strain RB51 vaccine confers protective immunity against virulent strains of *B. abortus* and *Brucella canis* in BALB/c mice. Microbiology. 2015;161(11):2137–2148. doi: 10.1099/mic.0.000170. 26341622

[120] Zhu L, Feng Y, Zhang G, et al. *Brucella suis* strain 2 vaccine is safe and protective against heterologous *Brucella* spp. infections. Vaccine. 2016;34(3):395–400. doi: 10.1016/j.vaccine.2015.09.116. 26626213

[121] Verger JM, Grayon M, Zundel E, et al. Comparison of the efficacy of *Brucella suis* strain 2 and *Brucella melitensis* rev. 1 live vaccines against a *Brucella melitensis* experimental infection in pregnant ewes. Vaccine. 1995;13(2):191–196. doi: 10.1016/0264-410X(95)93135-V. 7625115

[122] Gheibi A, Khanahmad H, Kashfi K, et al. Development of new generation of vaccines for *Brucella abortus*. Heliyon. 2018;4(12):e01079. doi: 10.1016/j.heliyon.2018.e01079. 30603712 PMC6307385

[123] Zhang J, Guo F, Chen C, et al. *Brucella melitensis* 16MΔhfq attenuation confers protection against wild-type challenge in BALB/c mice. Microbiol Immunol. 2013;57(7):502–510. doi: 10.1111/1348-0421.12065. 23647412

[124] Li Z, Zhang J, Zhang K, et al. *Brucella melitensis* 16MΔTcfSR as a potential live vaccine allows for the differentiation between natural and vaccinated infection. Exp Ther Med. 2015b;10(3):1182–1188. doi: 10.3892/etm.2015.2619. 26622461 PMC4533132

[125] Wang Z, Niu JR, Ui Wang XLe, et al. Evaluation of a *Brucella melitensis* mutant deficient in O-polysaccharide export system ATP-binding protein as a rough vaccine candidate. Microbes Infect. 2014;16(8):633–639. doi: 10.1016/j.micinf.2014.06.013. 25043564

[126] Costa LF, Cabello AL, Batista DFA, et al. The candidate vaccine strain *Brucella ovis* ΔabcBA is protective against *Brucella melitensis* infection in mice. Microbiol Immunol. 2020;64(11):730–736. doi: 10.1111/1348-0421.12850. 32965738

[127] Bao Y, Tian M, Li P, et al. Characterization of *Brucella abortus* mutant strain Δ22915, a potential vaccine candidate. Vet Res. 2017;48(1):17. doi: 10.1186/s13567-017-0422-9. 28376905 PMC5381064

[128] Czibener C, Del Giudice MG, Spera JM, et al. Delta-pgm, a new live-attenuated vaccine against *Brucella suis*. Vaccine. 2016;34(13):1524–1530. doi: 10.1016/j.vaccine.2016.02.025. 26899373

[129] Jain-Gupta N, Waldrop SG, Tenpenny NM, et al. Rough *Brucella neotomae* provides protection against *Brucella suis* challenge in mice. Vet Microbiol. 2019;239:108447. doi: 10.1016/j.vetmic.2019.108447. 31767087

[130] Ficht TA, Kahl-McDonagh MM, Arenas-Gamboa AM, et al. Brucellosis: the case for live, attenuated vaccines. Vaccine. 2009;27(Suppl. 4):D40–D43. doi: 10.1016/j.vaccine.2009.08.058. 19837284 PMC2780424

[131] Perkins SD, Smither SJ, Atkins HS. Towards a *Brucella* vaccine for humans. FEMS Microbiol Rev. 2010;34(3):379–394. doi: 10.1111/j.1574-6976.2010.00211.x. 20180858

[132] Zimmermann P, Curtis N. Factors that influence the immune response to vaccination. Clin Microbiol Rev. 2019;32(2):e00084. doi: 10.1128/CMR.00084-18. 30867162 PMC6431125

[133] Abdollahi A, Mansouri S, Amani J, et al. A recombinant chimera protein as a novel *Brucella* subunit vaccine: protective efficacy and induced immune response in BALB/c mice. Jundishapur J Microbiol. 2018;11(1):1–9. doi: 10.5812/jjm.12776.

[134] Ghasemi A, Jeddi-Tehrani M, Mautner J, et al. Immunization of mice with a novel recombinant molecular chaperon confers protection against *Brucella melitensis* infection. Vaccine. 2014;32(49):6659–6666. doi: 10.1016/j.vaccine.2014.09.013. 25240754

[135] Sadeghi Z, Fasihi-Ramandi M, Bouzari S. Evaluation of immunogenicity of novel multi-epitope subunit vaccines in combination with poly I: c against *Brucella melitensis* and *Brucella abortus* infection. Int Immunopharmacol. 2019;75:105829. doi: 10.1016/j.intimp.2019.105829. 31437796

[136] Delpino MV, Estein SM, Fossati CA, et al. Vaccination with *Brucella* recombinant DnaK and SurA proteins induces protection against *Brucella abortus* infection in BALB/c mice. Vaccine. 2007;25(37–38):6721–6729. doi: 10.1016/j.vaccine.2007.07.002. 17686554

[137] Abkar M, Fasihi-Ramandi M, Kooshki H, et al. Oral immunization of mice with Omp31-loaded N-trimethyl chitosan nanoparticles induces high protection against *Brucella melitensis* infection. Int J Nanomedicine. 2017;12:8769–8778. doi: 10.2147/IJN.S149774. 29263667 PMC5732559

[138] Maleki M, Salouti M, Shafiee Ardestani M, et al. Preparation of a nanovaccine against *Brucella melitensis* M16 based on PLGA nanoparticles and oligopolysaccharide antigen. Artif Cells Nanomed Biotechnol. 2019;47(1):4248–4256. doi: 10.1080/21691401.2019.1687490. 31718300

[139] Al-Halifa S, Gauthier L, Arpin D, et al. Nanoparticle-based vaccines against respiratory viruses. Front Immunol. 2019;10:22. doi: 10.3389/fimmu.2019.00022. 30733717 PMC6353795

[140] Leclerq S, Harms JS, Rosinha GMS, et al. Induction of a Th1-type of immune response but not protective immunity by intramuscular DNA immunisation with *Brucella abortus* GroEL heat-shock gene. J Med Microbiol. 2002;51(1):20–26. doi: 10.1099/0022-1317-51-1-20. 11803949

[141] Afshari H, Maleki M, Salouti M. Immunological effects of two new nanovaccines against *Brucella* based on OPS and LPS antigens conjugated with PLGA nanoparticles. Eur Polym J. 2020;139:110021. doi: 10.1016/j.eurpolymj.2020.110021.

[142] Hu XD, Yu DH, Chen ST, et al. A combined DNA vaccine provides protective immunity against *Mycobacterium bovis* and *Brucella abortus* in cattle. DNA Cell Biol. 2009;28(4):191–199. doi: 10.1089/dna.2008.0790. 19364278

[143] Chen B, Liu B, Zhao Z, et al. Evaluation of a DNA vaccine encoding *Brucella* BvrR in BALB/c mice. Mol Med Rep. 2019;19(2):1302–1308. doi: 10.3892/mmr.2018.9735. 30569140

[144] Jain S, Afley P, Dohre SK, et al. Evaluation of immunogenicity and protective efficacy of a plasmid DNA vaccine encoding ribosomal protein L9 of *Brucella abortus* in BALB/c mice. Vaccine. 2014;32(35):4537–4542. doi: 10.1016/j.vaccine.2014.06.012. 24950353

[145] Velikovsky CA, Cassataro J, Sanchez M, et al. Single-shot plasmid DNA intrasplenic immunization for the production of monoclonal antibodies. Persistent expression of DNA. J Immunol Methods. 2000;244(1–2):1–7. doi: 10.1016/S0022-1759(00)00244-1. 11033013

[146] Al-Mariri A, Tibor A, Lestrate P, et al. Yersinia enterocolitica as a vehicle for a naked DNA vaccine encoding *Brucella abortus* bacterioferritin or P39 antigen. Infect Immun. 2002;70(4):1915–1923. doi: 10.1128/IAI.70.4.1915-1923.2002. 11895955 PMC127831

[147] Senevirathne A, Hewawaduge C, Lee JH. Attenuated *Salmonella* secreting *Brucella* protective antigens confer dual-faceted protection against brucellosis and salmonellosis in a mouse model. Vet Immunol Immunopathol. 2019;209:31–36. doi: 10.1016/j.vetimm.2019.02.001. 30885303

[148] Bugybayeva D, Ryskeldinova S, Zinina N, et al. Development of human vectored brucellosis vaccine formulation: assessment of safety and protectiveness of influenza viral vectors expressing *Brucella* immunodominant proteins in mice and Guinea pigs. Biomed Res Int. 2020;2020:1438928. doi: 10.1155/2020/1438928. 33274194 PMC7695499

[149] Mohammadi E, Golchin M. High protection of mice against *Brucella abortus* by oral immunization with recombinant probiotic *Lactobacillus casei* vector vaccine, expressing the outer membrane protein OMP19 of *Brucella* species. Comp Immunol Microbiol Infect Dis. 2020;70:101470. doi: 10.1016/j.cimid.2020.101470. 32208191

[150] Lin GZ, Liu YZ, Cai KZ, et al. *Brucella abortus* L7/L12 ve BCSP31 Proteinlerini Birlikte Eksprese Eden Rekombinant Adenovirusların immunojenitesi. Kafkas Univ Vet Fak Derg. 2018;24(2):211–217. doi: 10.9775/kvfd.2017.18644.

[151] Bugybayeva D, Kydyrbayev Z, Zinina N, et al. A new candidate vaccine for human brucellosis based on influenza viral vectors: a preliminary investigation for the development of an immunization schedule in a Guinea pig model. Infect Dis Poverty. 2021;10(1):13. doi: 10.1186/s40249-021-00801-y. 33593447 PMC7886305

[152] Tabynov K, Sansyzbay A, Kydyrbayev Z, et al. Influenza viral vectors expressing the *Brucella* OMP16 or L7/L12 proteins as vaccines against *B. abortus* infection. Virol J. 2014;11(1):69. doi: 10.1186/1743-422X-11-69. 24716528 PMC3997475

[153] Sáez D, Fernández P, Rivera A, et al. Oral immunization of mice with recombinant *Lactococcus lactis* expressing Cu, Zn superoxide dismutase of *Brucella abortus* triggers protective immunity. Vaccine. 2012;30(7):1283–1290. doi: 10.1016/j.vaccine.2011.12.088. 22222868

[154] Cassataro J, Estein SM, Pasquevich KA, et al. Vaccination with the recombinant *Brucella* outer membrane protein 31 or a derived 27-amino-acid synthetic peptide elicits a CD4+ T helper 1 response that protects against *Brucella melitensis* infection. Infect Immun. 2005;73(12):8079–8088. doi: 10.1128/IAI.73.12.8079-8088.2005. 16299302 PMC1307072

[155] De la Rosa-Ramos MA, Arellano-Reynoso B, Hernández-Badillo E, et al. Evaluation of the goat cellular immune response to rBtuB-Hia-FlgK peptides from *Brucella melitensis*. Comp Immunol Microbiol Infect Dis. 2023;94:101944. doi: 10.1016/j.cimid.2023.101944. 36638645

[156] Nejad RB, Krecek RC, Khalaf OH, et al. Brucellosis in the Middle East: current situation and a pathway forward. PLoS Negl Trop Dis. 2020;14(5):e0008071. doi: 10.1371/journal.pntd.0008071. 32437346 PMC7241688

[157] Addis M. Public health and economic importance of brucellosis: a review. Public Policy Admin Res. 2015;5(7):2225–2972.

[158] Islam MA, Khatun MM, Werre SR, et al. A review of *Brucella* seroprevalence among humans and animals in Bangladesh with special emphasis on epidemiology, risk factors and control opportunities. Vet Microbiol. 2013;166(3–4):317–326. doi: 10.1016/j.vetmic.2013.06.014. 23867082

[159] Alewy Almashhadany D, Zefenkey ZF, Hassannejad S, et al. Milk borne brucellosis. In: Ibrahim SA, editor. Current issues and advances in the dairy industry. IntechOpen; 2023. doi: 10.5772/intechopen.109124.

[160] Di Bari C, Venkateswaran N, Bruce M, et al. Methodological choices in brucellosis burden of disease assessments: a systematic review. PLoS Negl Trop Dis. 2022;16(12):e0010468. doi: 10.1371/journal.pntd.0010468. 36512611 PMC9794075

[161] Herrera JAR, Thomsen ST, Jakobsen LS, et al. The burden of disease of three food-associated heavy metals in clusters in the Danish population–towards targeted public health strategies. Food Chem Toxicol. 2021;150:112072. doi: 10.1016/j.fct.2021.112072. 33610621

[162] Golshani M, Buozari S. A review of brucellosis in Iran: epidemiology, risk factors, diagnosis, control, and prevention. Iran Biomed J. 2017;21(6):349–359. doi: 10.18869/acadpub.ibj.21.6.349. 28766326 PMC5572431

[163] Franc KA, Krecek RC, Häsler BN, et al. Brucellosis remains a neglected disease in the developing world: a call for interdisciplinary action. BMC Public Health. 2018;18(1):125. doi: 10.1186/s12889-017-5016-y. 29325516 PMC5765637

[164] Dowdle WR. The principles of disease elimination and eradication. Bull World Health Org. 1998;76(Suppl. 2):22–25. 10063669 PMC2305684

[165] Murray J, Cohen AL. Infectious disease surveillance. Int Encycloped Public Health. 2016;2016:222–229. doi: 10.1016/B978-0-12-803678-5.00517-8.

[166] Roth F, Zinsstag J, Orkhon D, et al. Human health benefits from livestock vaccination for brucellosis: case study. Bull World Health Organ. 2003;81(12):867–876. 14997239 PMC2572379

[167] Dalrymple-Champneys W. Undulant fever a neglected problem. The Lancet. 1950;255(6602):429–435. doi: 10.1016/S0140-6736(50)90361-8.

[168] Wyatt HV. Brucellosis and Maltese goats in the Mediterranean. J Malt Hist. 2009;1(2):4.

[169] Riedel S. Biological warfare and bioterrorism: a historical review. Proc (Bayl Univ Med Cent). 2004;17(4):400–406. doi: 10.1080/08998280.2004.11928002. 16200127 PMC1200679

[170] Centers for Disease Control and Prevention. Risks from unpasteurized dairy products. Risk of exposure. Brucellosis. CDC; 2019. Available from: https://www.cdc.gov/brucellosis/exposure/unpasteurized-dairy-products.html

[171] Yuan W, Zhang M, Zou H, et al. Emergency response to occupational brucellosis in a pharmaceutical manufacturing enterprise. J Occup Health. 2018;60(5):404–409. doi: 10.1539/joh.2018-0089-CS. 30068856 PMC6176033

[172] Elbehiry A, Aldubaib M, Marzouk E, et al. The development of diagnostic and vaccine strategies for early detection and control of human brucellosis, particularly in endemic areas. Vaccines. 2023;11(3):654. doi: 10.3390/vaccines11030654. 36992237 PMC10054502

[173] Micoli F, Bagnoli F, Rappuoli R, et al. The role of vaccines in combatting antimicrobial resistance. Nat Rev Microbiol. 2021;19(5):287–302. doi: 10.1038/s41579-020-00506-3. 33542518 PMC7861009

[174] Behl T, Kaur I, Sehgal A, et al. Bioinformatics accelerates the major tetrad: a real boost for the pharmaceutical industry. Int J Mol Sci. 2021;22(12):6184. doi: 10.3390/ijms22126184. 34201152 PMC8227524

[175] Zhao C, Yang Y, Wu S, et al. Search trends and prediction of human brucellosis using Baidu index data from 2011 to 2018 in China. Sci Rep. 2020;10(1):5896. doi: 10.1038/s41598-020-62517-7. 32246053 PMC7125199

[176] World Bank. World Bank approves $82 million for prevention of zoonotic, endemic diseases in India. World Bank; 2023 [cited 2023 Aug 23]. Available from: https://www.worldbank.org/en/news/press-release/2023/05/10/world-bank-approves-82-million-for-prevention-of-zoonotic-endemic-diseases-in-india

